# Comparison of researchers’ impact indices

**DOI:** 10.1371/journal.pone.0233765

**Published:** 2020-05-29

**Authors:** Samreen Ayaz, Nayyer Masood

**Affiliations:** Department of Computer Science, Capital University of Science & Technology, Islamabad, Pakistan; UNSW, AUSTRALIA

## Abstract

Researchers contribute to the frontiers of knowledge by establishing facts and reaching new conclusions through systematic investigations, and by subsequently publishing the outcomes of their research findings in the form of research papers. These research publications are indicative of researchers' scientific impact. Different bibliometric indices have been proposed to measure the impact or productivity of a researcher. These indices include publication count, citation count, number of coauthors, h-index, etc. The h-index, since its inception, has been ranked as the foremost impact indicator by many studies. However, as a consequence of the various short comings identified in h-index, some variants of h-index have been proposed. For instance, one dimension which requires significant attention is determining the ability of exceptional performers in a particular research area. In our study, we have compared effectiveness of h-index and some of its recent variants in identifying the exceptional performers of a field. We have also found correlation of h-index with recently proposed indices. A high correlation indicates same effect of these indices as of h-index and low correlation means these indices make non-redundant contribution while ranking potential researchers of a field of study. So far, effectiveness of these indices has not been explored/validated on real data sets of same field. We have considered these variants/modifications of h-index along with h-index and tested on comprehensive data set for the field of Computer Science. The Award winners’ data set is considered as the benchmark for the evaluation of these indices for individual researchers. Results show that there is a low correlation of these indices with h-index, and in identifying exceptional performers of a field these indices perform better than h-index.

## 1. Introduction

Different bibliometric methods are used for evaluating scientist’s research impact. Hirsch [1] defines h-index as, “an author has an index h if at least h number of his/her publications have h citations each”. h-index is widely adopted by research community/evaluators. The reason of this adoption is that it is easy to compute, quantity and quality are simultaneously considered and above all one number quantifies the research output effectively. These advantages are complemented by some drawbacks like self-citations, dependence on scientific area, less sensitive to highly cited papers and dependence on length of scientist’s career etc.

Beside other shortcomings a major shortcoming of h-index is that it is field dependent. h-index precludes a comparison of scientists from different fields. Different fields have different patterns for number of citations/ publications as highlighted in many studies [1, 2, 3]. H-index based comparison of researchers from different fields is both unrealistic and unfair. Keeping in view h-index’s incapacity to compare scientists from across different fields of study, different variants of h-index have been proposed [4, 5, 6]. Dienes mentions a serious mathematical incompleteness/deficiency in the basic definition of h-index. This deficiency projects a prejudiced approach in the comparison of scientists from different fields of study. Due to this deficiency, it doesn’t balance the effect of citations and publications. Dienes proposed steps to balance the effect and presented idea of completing h-index [5].

To address different drawbacks of h-index, Kinouchi et al. recently proposed to add one level in the h-index calculations. Kinouchi et al. mentions that because of narrow dynamic range of h-index, it is unable to differentiate high impact authors having less number of publications from relatively low impact authors. Other identified drawbacks include: h-index is dependent on scientific area, its susceptibility to self-citations and an ambiguity in relationship with qualitative measures like awards, scientific prizes etc. [7]. Moreover h-index cannot measure the influence of a person in the community at large. To address these shortcomings Kinouchi et al. have proposed a new centrality index called K-index. K-index considers the network of papers and authors [7]. According to authors, K-index addresses many drawbacks of h-index. It is not contingent on number of publications, it not only addresses the issue of self-citations, but also has large classification range. Moreover, it is able to detect scientific counterfeits, also it is better correlated with scientific awards and it can be easily calculated using Web of science (WOS). Authors have claimed that K-index has so many advantages over h-index, but considering a small sample of researchers from field of Physics makes it debatable. This necessitates checking the validity of claims for a comprehensive data set of authors across different fields and evaluating its effectiveness for scientific prizes in comparison with h-index. To check the validity of claims about completing-h and K-index, this study proceeded in three steps: first, a calculation of Spearman and Pearson’s correlation coefficients for these indices was carried, second, an evaluation of the performance of these indices against award winner’s benchmark was evaluated, and in the third step, a slight variation in the rankings of authors is proposed and an evaluation of its results against these indices. For this purpose, a comprehensive data set ArnetMiner available at https://cn.aminer.org/billboard/aminernetwork was considered in the field of Computer Science. It is pertinent to foreground here that in the absence of a benchmark for evaluating the effectiveness of an index in identification of excellent performers, we considered Computer Science award winners as our benchmark.

Our findings indicate that all of these indices are highly correlated as far as Pearson Correlation is concerned. However when Spearman rank correlation was applied, the correlation among ranked lists was relatively low. In terms of effectiveness of indices, we have found that completing-h and K-index succeeded in bringing highest number of award winners in top ranks. Thus, having found this, we posit our conclusion that these indices perform comparatively better than h-index. We infer from this study that the investigated indices must have effectively removed the stated deficiencies of h-index.

## 2. Literature review

İn 2005 Hirsch proposed a measure to quantify the scientific impact of researchers [1]. This is the h-index measure. Hirsch elaborated a number of advantages of h-index in comparison to other bibliometric indicators which include: number of publications, number of citations, citations per paper and number of significant papers. It is pertinent to remind here that h-index was no less than a revolution in the field of researcher’s evaluation and bibliometrics. World adopted it instantly and nowadays h-index is one of the leading evaluation criteria for researchers.

Introduction of h-index triggered a new research front. This research resulted in a number of extensions and variants of h-index. Many researchers have compared and evaluated different variants/extensions of h-index and other bibliometric indicators [8, 9, 10, 11, 12].

A few other researchers have emphasized the use of normalized values instead of direct calculation of h-index. Bornmann & Leydesdorff have asserted on replacement of h-index by some normalized indicators. They have emphasized that a reasonable alternative to the h-index is to count the top cited papers in the corresponding fields and the top-10% most-highly cited papers can be used for measuring excellence [13].

Addressing another drawback of h-index a time dependent dynamic h-type index was proposed by Rousseau & Ye [14]. The foremost competence of this study rests in being able to tally the most recent changes in the research performance of an author. According to the proposers, it is much more advantageous for hiring purposes than the h-index, as this index explicitly indicates the recent achievements of researchers unlike h-index that covers the life time of a researcher.

Radicchi & Castellano also emphasized that even citations cannot be used directly to compare the researchers from different fields or years [15]. They have asserted that normalization of citations by number of publications has positive effects. It reduces bias and is preferably recommended when researchers from different fields are compared.

Similar approach is adopted by Iglesias & Pecharromán [16]. Authors have emphasized that simple h-index is meaningless in comparing researchers from different fields, as different fields have different citation habits. They have also proposed that average number of citations per paper for any scientific field should be used for normalization purposes while comparing researchers across the fields.

van Zyl & van der Merwe presented a proposal to overcome the field dependent deficiency of h-index emphasizing that citations per publication for different fields can be used for normalization of h-index across the fields [17]. Schreiber considered 20 variants of h-index based on the criteria of considering or not considering highly cited papers. Small sample of 26 physicist is considered and correlation among all the indices were calculated. Advantages and disadvantages of these indices were discussed. Interestingly Most of the indices were found to be highly correlated [18].

h-index has proven to be incompatible to comparison of scientists in different domains since originally it was proposed for individual evaluation. Dienes argued that community role should be considered while evaluating an author. He points out that there is an intrinsic deficiency in basic definition of h-index. With the inclusion of community factor this deficiency can be overcome/removed and cross domain comparison is also possible. Dienes has proposed to add one step in basic definition of h-index [5]. He agrees with basic philosophy of h-index i.e. to combine effect of two important measures, citations and publications. But, the deficiency responsible for the variation in usefulness of h-index for some fields more than the others needs to be removed.it is applicable also for scenarios where h-index merely depicts number of paper or citation counts which differ by a large magnitude. In extreme situations it is unable to balance the effect of paper count and citation count. Thus in order to remove /overcome this deficiency inclusion of community factor for researchers has been proposed.

Since the researchers has not assessed/calculated the impact of completing-h on different fields. So, there is need to evaluate usefulness of completing-h on real data set of scientists of different fields. Ayaz & Afzal have evaluated completing-h in the discipline of Mathematics [19]. Prestigious awards given in the field of Mathematics are considered as benchmark. According to their findings, completing-h has outperformed h-index and g-index. Completing-h succeeded in bringing more award winners in top ranks than h-index and g-index.

A newly proposed K-index [7] addresses many shortcomings of h-index, but it is not checked on any comprehensive data set, but only for small sample of physicists.

The above mentioned two recently proposed author ranking indices i.e. completing-h and K-index assure to fulfil the deficiencies of the h-index. However, there is no study that evaluates them on a common comprehensive data set. Motivated by this fact, this study verifies the promises/claims by the two indices and compares their ranking using correlation, awards benchmark and a comprehensive data set that relates to the field of Computer Science.

## 3. Methodology

In this research work, we have considered comprehensive data set of researchers for the field of Computer Science, and calculated h-index, K-index and completing-h indices for researchers. The objective is to evaluate which of these prominent indices better reflects the impact of researchers. For this purpose, we have considered award winners of prestigious awards in the field of Computer Science as standard and compared the indices’ rankings against it. In this section we have discussed in detail all the steps taken to perform this comparison/analysis.

### 3.1. Data set

**Fig 1:**
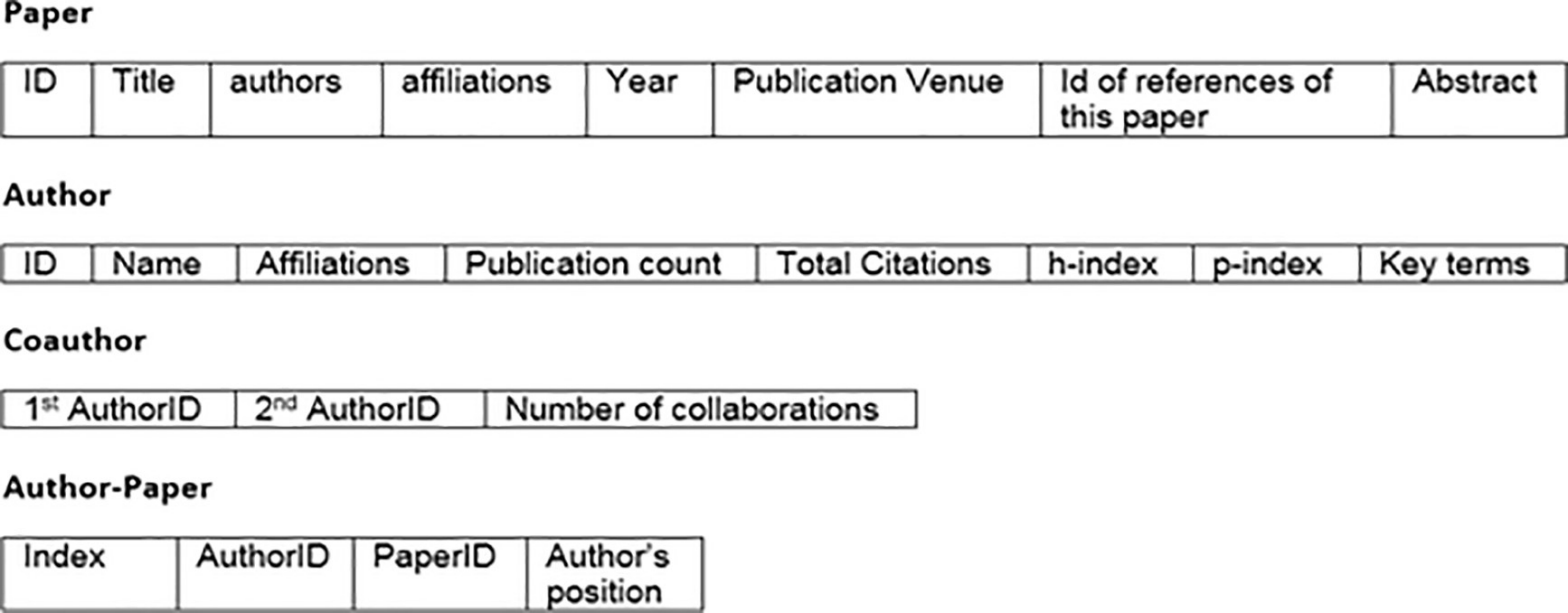
Relations & their attributes.

ArnetMiner is a system for extracting and mining academic social networks [20]. The ArnetMiner data set covers publications record for the field of Computer Science, collected from ACM and DBLP data repositories [20]. The tables/entities which are included in the data set are papers, authors, coauthors and author-papers. Author-paper table includes primary keys of authors(ID as authorID) and papers(ID as paperID) as foreign keys, so author and papers relationship is related through this table. Initially, the data set had 2,092,356 publications and 8,024,869 citations between them, also record of 1,712,433 authors and 4,258,615 collaboration relationships between authors. Data set have publications record till 2014. Detail of attributes is given in Fig 1.

#### Author disambiguation

According to Tang et al., author disambiguation step was performed during the collection of this data set [20] but during data analysis we found that the author duplication problem was still there. There were number of occurrences of authors having same name but different IDs. Sometimes they were actually different but in lot of cases the authors were same but were given different IDs. After analysis, we have come to know that affiliation of an author is a very important factor in identifying whether two authors with same names are actually same or different.

First, we identified all author names having more than one occurrences in data set. Then we examined their respective affiliations; authors with the same name and having same affiliations were considered to be duplicate, whereas those having same name but different affiliations were treated as different authors. After performing this basic level of deduplication, we were left with 1,529,733 authors whereas originally we had 1,712,433 authors.

#### Sampling

It would require a lot of time and resources to compute indices from such a huge population. As an example, consider the case of computing citations of papers. Total number of references in this data set is 9,268,353, so to compute citations of a single paper from this data set, it would require 9,268,353 comparisons from references table, whereas total number of papers in this data set is 2,092,356. Now to compute citations for all these papers would require thousands of millions of comparisons. In order to handle this issue, we applied stratified sampling technique. Detail of considering samples and estimating standard error with confidence interval of 95% is given in supplementary material (S1 Appendix). Data statistics of the sample data set we considered is given in Table 1 and is available at https://github.com/samreenayaz/Dataset.

**Table 1:** Statistics of sample data set.

	Count
Number of publications	236,416
Number of Authors	76,750
Number of Citations	3,039,169
Number of award winners	1,994
Number of award winners after removing duplication	1,443
Number of award winners matched with complete Arnetminer data set	1,206
Number of award winners found in sample data set	47

#### Award winners

Our literature review reveals that there is no standard benchmark data set available to evaluate the performance or effectiveness of different indices. There are some studies in which award winners or Nobel Prize winners of respective fields are used as benchmark [21, 22]. According to [23] high profile scientists (e.g. Nobel laureates and members National of Academy of Sciences) generally score higher h index values. Thus according to our proposal/assumption, the index which succeeds in bringing award winners in top ranks is the most successful index. Following the Mathematics awardees benchmark dataset [19], we decided to use the awardees dataset for the field of Computer Science as benchmark. For this purpose, we considered prestigious awards given in this field ranging from year 1966 to 2014. Statistics for authors and award winners’ data that we collected are given in Table 1. In total, we have worked on 24 awards which are awarded by two well-known organizations in CS i.e. ACM and IEEE. Some of the awards that we have considered include ACM Fellow, IEEE Technical achievement Award and Turing Award. Complete list of the awards and names of award winners are given in S2 and S3 Appendices respectively.

Initially, 1,994 award winners were considered, but there are some researchers who have been awarded more than one prizes. Duplicate entries for such award winners were removed. After removing duplication, we were left with 1,443 award winners. Out of these, 1,206 award winners matched with Arnetminer data set as a whole, whereas 47 award winners were matched with our sample data set.

### 3.2 Calculation of indices

For all the authors in sample data set we have calculated h-index, completing-h and K-index. Research community is quite familiar with the calculation of h-index. Here we have discussed in detail, how to calculate completing-h and k-index.

#### Completing-h

**Fig 2:**
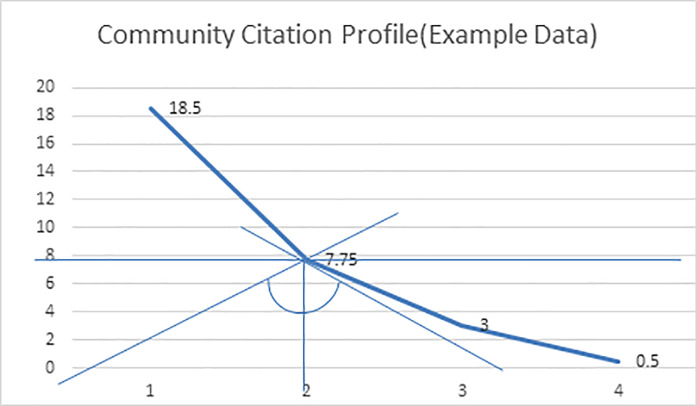
Community citation profile (Example data).

According to Dienes, number of publications and citations are two different entities. If the magnitude of these two values highly differs, there would be no balance in the calculated h-index. As h-index combines two different values, hence there should be a step to balance these two entities [5]. He illustrates that we should consider a curve between number of citations and rank of publications on the basis of citations value. The point at which the curve intersects these two values, that is the point at which citations and rank are same. The tangent line at this point should always be perpendicular to the line originating from origin shown in Fig 2. Then we can say that there is true balance between number of citations and number of publications.

The difference between the calculation of h-index and completing-h is the introduction of conversion factor. To calculate this conversion factor, we have to consider the citation record of the community. Citation records of all the researchers are sorted in descending order separately as shown in Table 2 where we have presented, as an example, publication and citation records of 4 authors. First column contains the author Id, second column gives the paper Id and the last column contains the citation count of the papers in the descending order of citation count for an individual author. After this, the citation count of top level paper of all authors is summed and average for these papers is calculated as shown in Table 3. For example, the sum of top level papers of four authors of Table 2 is 74 and their average is 18.5. Likewise, the citation sum and average for all four levels is calculated. Then a curve showing the trend of this citation record is shown in Fig 2.

**Table 2:** Citation profile of authors (Example).

Author_Id	Paper_ID	CitationCount
**1**	1.1	24
**1**	1.2	15
**1**	1.3	5
**2**	2.1	7
**2**	2.2	1
**3**	3.1	30
**3**	3.2	10
**3**	3.3	5
**3**	3.4	2
**4**	4.1	13
**4**	4.2	5
**4**	4.3	2

**Table 3:** Community citation profile (Example).

Citations Ranking	Total Citations	Average Citations
**1**	74	18.5
**2**	31	7.75
**3**	12	3
**4**	2	0.5

According to Dienes, h-index lacks this intrinsic point in its definition that is to find a location on this curve where the slope of the tangent line is equal and opposite to the slope of a line connecting that location to the origin. It means the two angles in figure should be equal [5]. The conversion factor for this field will be calculated as ca = cotθ. In our example data this angle is approximately 60 degree and cotangent of this angle is 0.57735. Hence the conversion factor for our example data population is ca = 0.57735.

To calculate h-index we rank all the publications of an author in citation wise descending order. But here is again one extra step in completing-h. To compute completing-h we will multiply all the rankings for individual authors by the conversion factor as shown in Table 4 for our example data set of Table 2. Rest of the procedure is same as for calculating h-index.

**Table 4:** Complete-h and h-index calculated for example data.

*AuthorID*	*Paper_ID*	*Papers’ Rank*	NewRank = Rank*0.577355	*CitationCount*	*Authors’ Index Values*	
					Complete-h	h-index
*1*	1.1	1	0.57735	24		
*1*	1.2	2	1.1547	15		
*1*	1.3	3	1.73205	5	3	3
*2*	2.1	1	0.57735	7		
*2*	2.2	2	1.1547	1	1	1
*3*	3.1	1	0.57735	30		
*3*	3.2	2	1.1547	10		
*3*	3.3	3	1.73205	5	3	3
*3*	3.4	4	2.3094	2		
*4*	4.1	1	0.57735	13		
*4*	4.2	2	1.1547	5		
*4*	4.3	3	1.73205	2	3	2

With reference to the actual dataset considered in this study, we have devised community citation profile, then after mapping curve of this citation profile we have identified the angle and i.e. 16.50, hence the conversion factor found for this data set is ca = 3.375943.

#### K-index

According to the definition of K-index, “If a researcher has k citing papers, each one with at least k citations, then his K-index is k” [20]. This index considers citations from one level deeper than h-index. To calculate K-index, consider all the papers citing the publications of an author. Sort those papers in descending order on the basis of their citations. Now the researcher’s K-index would be n, number of publications having cited by papers having at least n citations each. Calculations of K-index are further clarified in Tables 5 and 6, considering the example data of Table 2. In this example value of h-index for authorID 1 is’ 2’ and value of h-index for authorID 2 is ‘2’ as well. Whereas K-index value for authorID 1 is ‘3’ and for authorID 2 is ‘5’.

**Table 5:** Citation record of 2 authors from Table 2.

AuthorID	PaperID	Citations	Cited_by (paperID)	Citation Count
**1**	1.1	2	3.1	30
**1**	1.1		3.2	10
**1**	1.2	2	4.1	13
**1**	1.2		4.3	2
**1**	1.3	1		
**2**	2.1	3	3.1	30
**2**	2.1		1.1	24
**2**	2.1		3.2	10
**2**	2.2	2	4.1	13
**2**	2.2		1.3	5

**Table 6:** Calculation of K-index.

*AuthorID*	*No.*	*Sorted Citation count*	*K-index*
*1*	1	30	
*1*	2	13	
*1*	**3**	**10**	
*1*	4	2	
*1*	5		3
*2*	1	30	
*2*	2	24	
*2*	3	13	
*2*	4	10	
*2*	**5**	**5**	5

### 3.3 Correlation among the values of indices

Spearman and Pearson correlation coefficients are determined for these three indices. The purpose of finding correlation is to check how much similar results these indices produce. Spearman correlation is particularly useful, as it would find correlation in ranked lists of authors i.e. it would evaluate whether the ranked lists acquired from different indices are similar or different.

### 3.4 Occurrences of award winners in rankings of authors

To evaluate the performance of these three indices, we have used awardees as benchmark. The idea is to rank the authors in descending order on the basis of values of these indices and then verify which index succeeds in bringing highest number of award winners in top ranks. First, we have made separate ranked lists of authors on the basis of their completing-h, K-index and h-index values. We have marked all the award winners found in our data set and their position in these ranked lists. We have identified how many award winners are found in top 10% of these ranked lists, then in next 10–20%, followed by 20–30%, 30–40%, 40–50% and then below 50% for all the ranked lists.

### 3.5 K_S_-index: A variation of K-index

In this section, we are proposing a variation of K-index, named K_S_-index, that we believe is a better indicator of authors’ scientific impact. It is established in scientific community that researchers having high h-index have more impact or are scientifically highly recognized. Our proposal is that papers cited by authors having high h-index value should be considered as more significant/influential papers in the domain. In the K-index, citations of the citations are considered to calculate the author’s index. In our proposed K_S_-index, we have considered the performance/h-index of authors who have cited certain paper by an author under consideration i.e., rather than the citation of the paper, we have considered the h-index of the authors of the citations. Idea is, to assess the researchers’ performance/calibre/scientific social recognition by considering the impact of researchers who cite their papers. Hence, we rank authors on the basis of other author’s impact who cite their work. We would like to mention that we have also considered self citations factor and filtered out self citations.

To explain the calculation of K_S_-index, we have considered as an example, citation records of 3 authors in Table 7. In this table, we have represented authors as authorID, the papers written by these authors (paper_ID) and the papers which have cited these papers (Cited_by_Paper_ID). For example, author 1 and 2 co-authored a paper, that is, P1, and this paper has been cited by three papers which are P8, P12 and P4. Table 7 then contains the author IDs of the citing papers. In Table 8, the h-index of all the authors from Table 7 are given.

**Table 7:** Citation record of *3* authors.

AuthorID	Paper_ID	Cited_by_Paper_ID
**1**	P1	P8,p12,p4
**1**	P2	P9,P4,P10
**1**	P3	P4
**2**	P1	P8,p12,p4
**2**	P4	P9,P5
**3**	P5	p8
**3**	P4	P9,P5
**3**	P6	P2,P5
**3**	P2	P9,P4,P10
**4**	P8	
**5**	P8	
**6**	P9	
**7**	P9	
**8**	P9	
**9**	P9	
**9**	P10	
**10**	P10	
**10**	P12	

**Table 8:** h-index of all authors (Table 7).

Authorid	Hindex
**1**	2
**2**	2
**3**	2
**4**	23
**5**	1
**6**	7
**7**	6
**8**	9
**9**	12
**10**	11

The calculation of K_S_-index has been explained in the Table 9 below. First column contains the *AuthorID*, and the second column contains the *PaperID* of the papers by an author. The third column *Cited_by_paper* contains the IDs of the papers citing the papers of an author, followed by *Cited_by _authorid*, the IDs of the authors of papers citing a paper. Then the h-index of the authors is given in *Cited_by_authorid_hindex* and their corresponding sorted order in Sorted_order. Each author’s record is sorted in descending order on the basis of *Cited_by_authorid_hindex*. Now K_S_-index would be n number of publications, having cited by authors with at least n h-index each. The K_S_-index for authors 1, 2 and 3 is 6, 5 and 7 respectively, given in last column of Table 9.

**Table 9:** Calculation of K_S_-index.

Authorid	Paper_ID	Cited_by_paper	Cited_by _authorid	K_S_-index	Sorted order	Cited_by _authorid_hindex
1	P1	P8	4	1	23	
1	P2	P9	9	2	12	
1	P2	P10	9	3	12	
1	P1	P12	10	4	11	
1	P2	P10	10	5	11	
1	P2	P9	8	**6**	**9**	**6**
1	P2	P9	7	7	6	
1	P1	P4	3	8	2	
1	P3	P4	3	9	2	
1	P2	P4	2	10	2	
1	P3	P4	2	11	2	
1	P1	P8	5	12	1	
2	P1	P8	4	1	23	
2	P4	P9	9	2	12	
2	P1	P12	10	3	11	
2	P4	P9	8	4	9	
2	P4	P9	7	**5**	**6**	**5**
2	P1	P4	3	6	2	
2	P4	P5	3	7	2	
2	P1	P8	5	8	1	
3	P5	P8	4	1	23	
3	P4	P9	9	2	12	
3	P2	P9	9	3	12	
3	P2	P10	9	4	12	
3	P2	P10	10	5	11	
3	P4	P9	8	6	9	
3	P2	P9	8	**7**	**9**	**7**
3	P4	P9	7	8	6	
3	P2	P9	7	9	6	
3	P2	P4	2	10	2	
3	P6	P2	1	11	2	
3	P5	P8	5	12	1	

## 4. Results and discussions

Authors are ranked on the basis of different indices and their rankings are evaluated from different perspectives. In section 4.1 and 4.2 we are comparing the previous indices, that is, h-index, k-index and completing-h index, whereas in section 4.3 we have discussed the proposed K_S_-index.

### 4.1. Correlation among the values of indices

**Fig 3:**
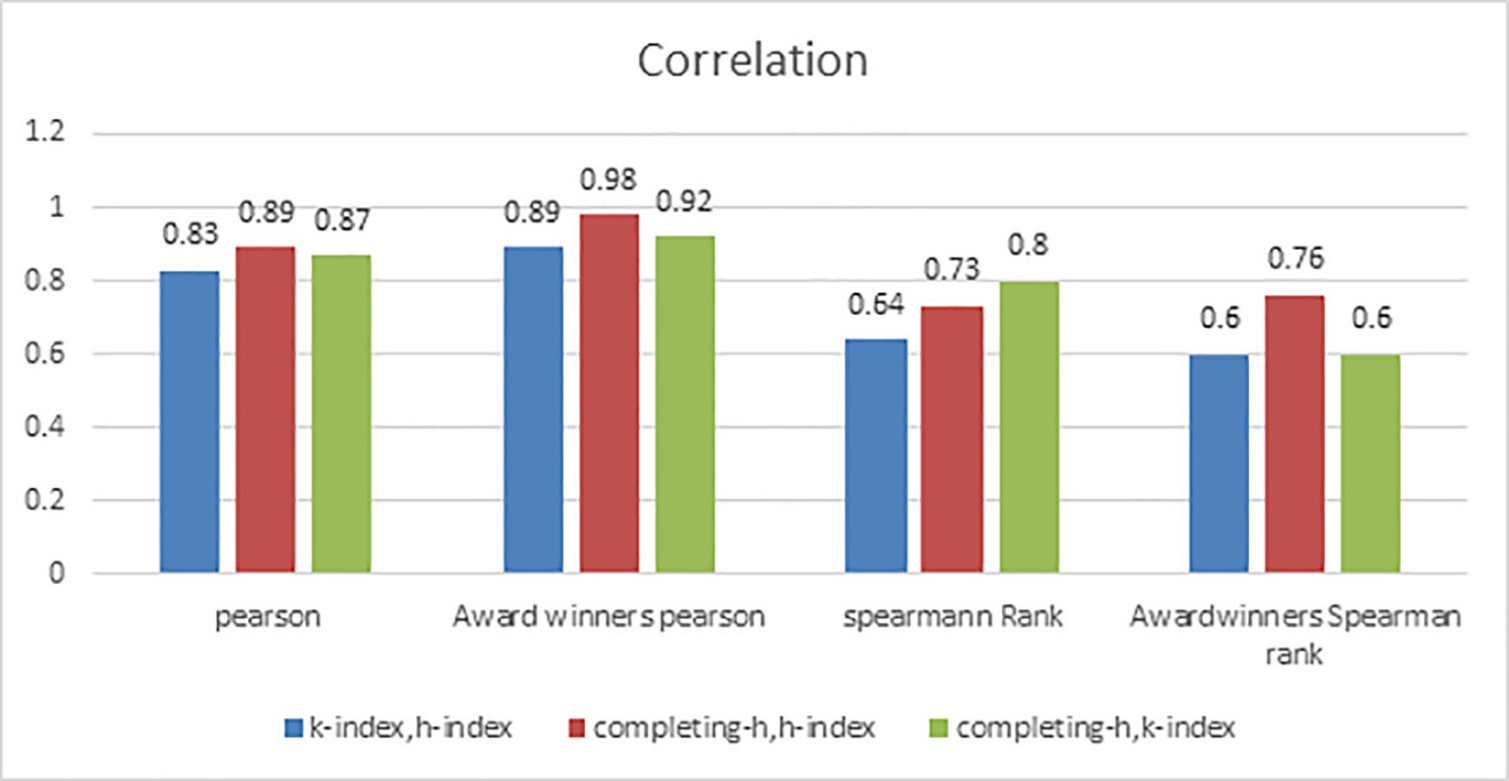
Correlation among indices.

Results of correlation among three indices are presented in Fig 3. In this figure, we have shown Pearson correlation and Spearman correlation, among all the authors and among only award winners. It is quite clear that Pearson correlation coefficient has high values whereas Spearman ranked correlation coefficient has somewhat lower values. It implies, that although indices are highly correlated, but the ranked lists obtained on the basis of these indices are moderately correlated. Actually, Pearson correlation represents linear relationship between two variables, whereas Spearman rank correlation measures monotonic relationship which can be nonlinear [24]. It is quite interesting to note that Spearman rank correlation of sample data set and award winner’s data set between K-index and h-index is low. It implies that rankings for h-index and K-index deviate.

### 4.2. Ranking of authors

To further evaluate the rankings by these indices we have compared against award winner’s data. Authors are ranked according to their completing-h, K-index and h-index values separately. From these rankings we have evaluated the occurrence of award winners in these ranked lists. First, we have evaluated what percentage of award winners are found in top 10% of these ranked lists, then next 10% i.e. 10–20% and so on till 50%, and then below 50%.

**Fig 4:**
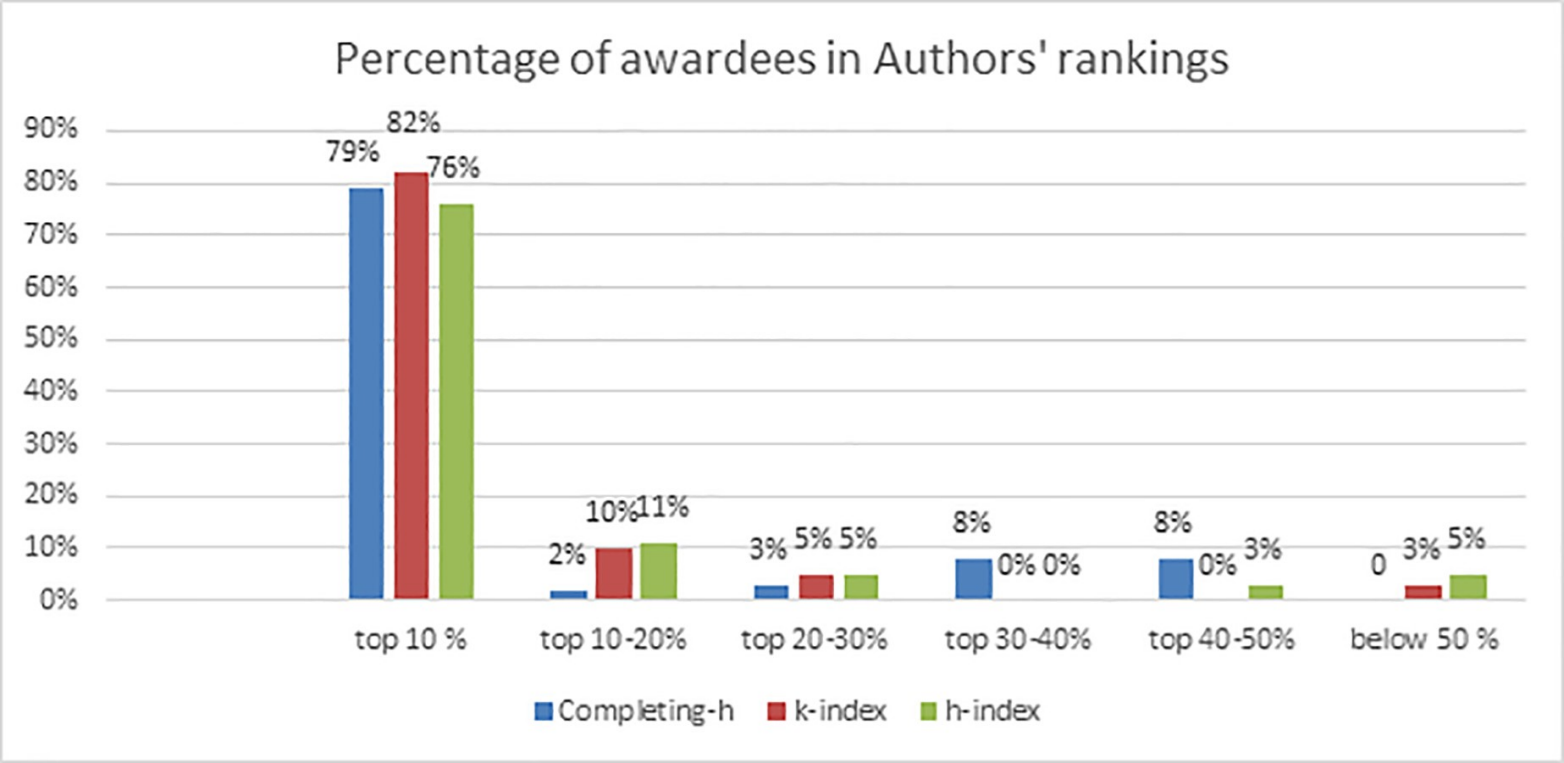
Occurrence of award winners in ranked lists.

These are shown in Table 10 and Fig 4. From Table 10 it is quite clear that all the indices have succeeded in identifying high percentage of award winners in top ranks, i.e. top 10 percent. For example of all the award winners found in our sample data set, completing-h succeeded in bringing 79% of award winners in top 10% researchers whereas 82% were brought by K-index and 76% by h-index. Though K-index seems most successful but in broader picture performance of h-index is also good. Only completing-h has high percentage of authors in low ranks. Hence K-index performs overall better than other indices in identifying exceptional performers and getting them in top ranks.

**Table 10:** Occurrence of award winners in ranked lists.

*Authors’ rankings*	*Awardees Percentage*		
	Completing-h	K-index	h-index
*top 10%*	79%	82%	76%
*top 10–20%*	2%	10%	11%
*top 20–30%*	3%	5%	5%
*top 30–40%*	8%	0%	0%
*top 40–50%*	8%	0%	3%
*below 50%*	0%	3%	5%

### 4.3. Impact of K_S_-index

To measure impact of citing authors, we have proposed and evaluated a variation in K-index. As described above, K_S_-index considers the h-index of citing authors to evaluate an author’s performance. Correlation of this proposed variation with h-index, completing-h and K-index was calculated and then the occurrence of award winners was checked in the list ranked accordingly.

**Fig 5:**
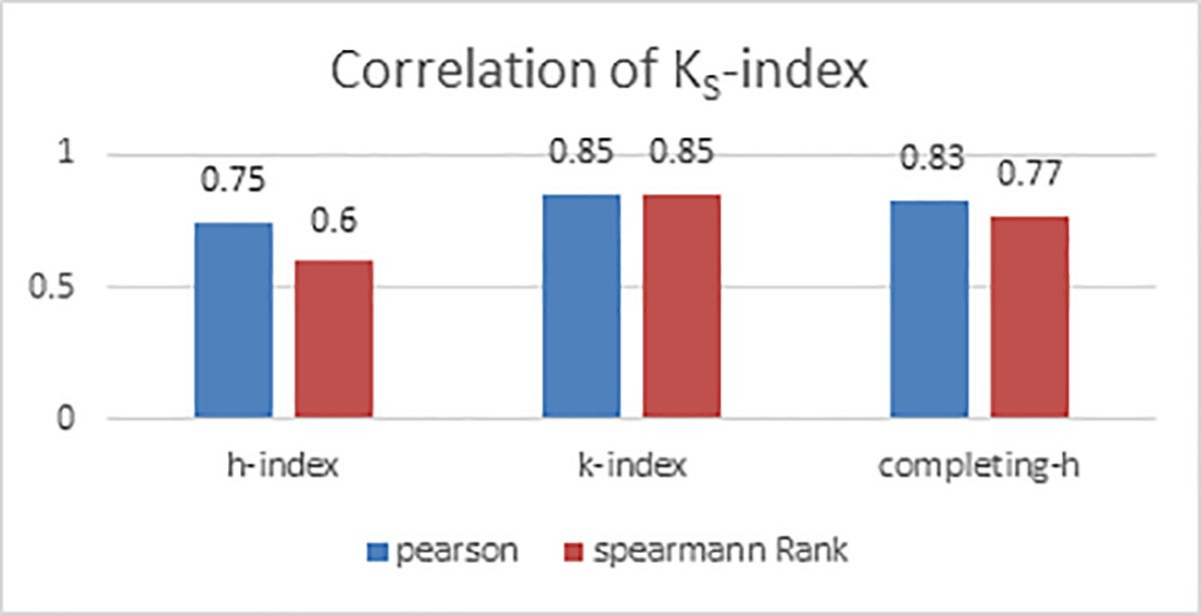
Correlation of K_S_-index.

As shown in Fig 5, K_S_-index has highest Pearson correlation with K-index and high correlation with completing-h. But has moderate value of Spearman rank correlation with completing-h and h-index. We can infer from this that the rankings of authors obtained by K_S_-index are somehow different from h-index and completing-h.

**Fig 6:**
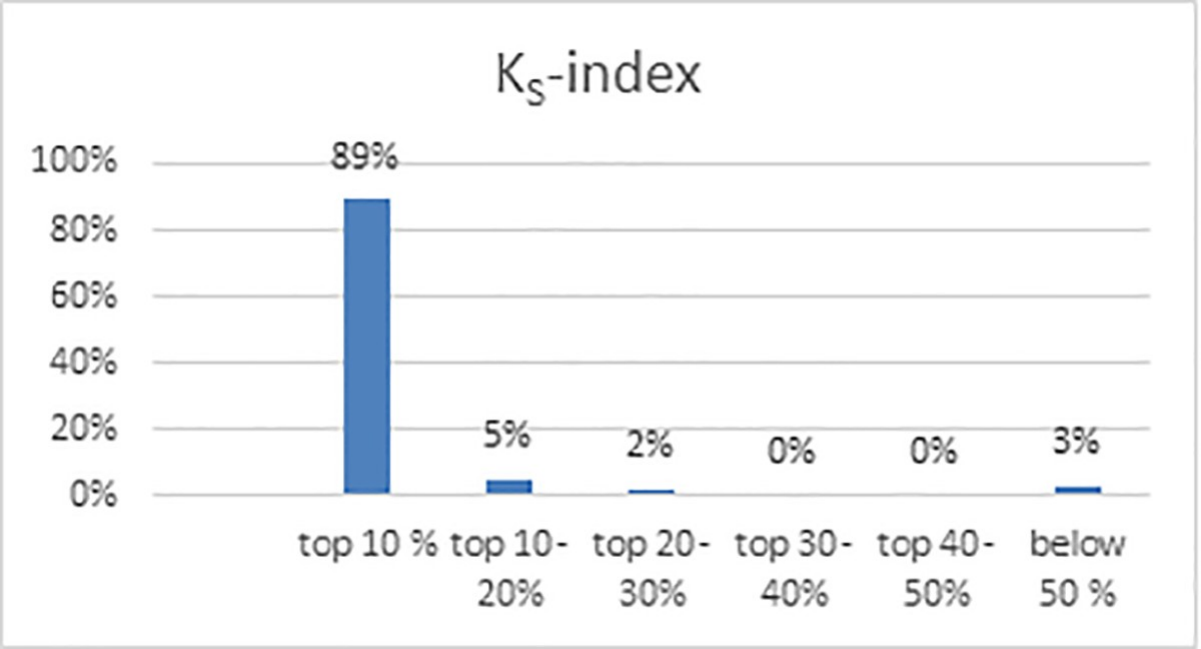
Occurrences of award winners in ranked list of K_S_-index.

Moreover, the K_S_-index succeeded in bringing highest number of award winners in top ranks. As shown in Fig 6, 89% of award winners lie in top 10% and 97% are in top 30% ranked lists.

From results it is quite evident that three new indices outperform h-index. But we should consider the complexity in calculations of these indices as compared to h-index. h-index is relatively simple to compute whereas these three indices require a lot of computation. Moreover when we consider completing-h, conversion factor for a community belong to the time/era for which it is calculated. Also we require citation data for whole community to calculate completing-h conversion factor. These factors should also be considered along with better results of these indices in comparison with h-index.

## 5. Conclusion

The objective of the study was to measure the effectiveness / impact of h-index and two newly proposed indices in identifying the exceptional performers/researchers in the field of research, especially in the field of Computer Science. One of the indices i.e. K-index has not been evaluated earlier while completing-h remains limited to the discipline of Mathematics. We have considered authors from the field of computer Science and award winners for this field as benchmark to evaluate the performance of considered indices. It was observed that Pearson correlation is high between these indices. Whereas Spearman rank correlation is low between the indices, which specifies the differences in rankings of authors based on the values of indices.

These indices are also analyzed on the foundation of rankings generated on the basis of the values of these indices. It was assumed that the index which will succeed in bringing highest number of award winners in top ranks will be considered as most successful in identifying the exceptional researchers. From the results it was observed that K-index is the index which brought highest number of award winners in top ranks followed by completing-h. But completing-h has high percentage of authors in low ranks as well. In conclusion, overall K-index performs better than h-index and completing-h.

From this study we have concluded that K-index is much better suited for the field of Computer Science than h-index. In another study completing-h outperformed h-index for the field of Mathematics and it claims to straighten the deficiency in h-index definition which made h-index field dependent. K-index assertion of covering the social recognition of an author, brought better results proving that K-index precedes h-index in performance. Hence, this necessitates further exploration and study of these indices for other fields and for finding certain metrics which can be used for fields across board.

## Supporting information

S1 Appendix (DOCX)

S2 Appendix (DOCX)

S3 Appendix (DOCX)
