# Supplementary material for: Comparison of researchers’ impact indices
Source: PLoS One. 2020 May 29;15(5):e0233765. doi: 10.1371/journal.pone.0233765 (PMC7259586; doi:10.1371/journal.pone.0233765)
Supplement: S1 Appendix — (DOCX) [file pone.0233765.s001.docx]

Appendix A

A sample should be able to represent the whole population and it should be un biased. Random sampling is done to avoid biasness in the sample selection. Considering the nature of problem we have adopted stratified sampling technique. In stratified sampling, population is divided into groups called strata, related cases are grouped together. Within/from each group, sample is randomly selected.

Initially, we have divided the entire population on the basis of h-index value. A large number of authors have the h-index value of 0 in this data set as evident from the table A_1_. In table A_1_, we have shown, in how many strata we have divided the whole population.

Table A_1_:

| h-index | Number of Authors | |
| --- | --- | --- |
|  | **Population** | **Randomly chosen Sample** |
| 0 | 850857 | 41403 |
| 1 | 497016 | 24840 |
| 2 | 82718 | 5265 |
| 3 | 38195 | 2130 |
| 4 | 20580 | 1097 |
| greater than 4 | 40375 | 2015 |
| Total | 1529733 | 76750 |

A good random sample should have more than 30 number of records and should be less than 10% of entire population. So we have considered sample size of approximately 5% of entire population. After dividing the whole population in 6 different stratum, on the basis of h-index value, we have randomly selected 5% records from each stratum.

A good random sample’s point estimate (mean in this case) should satisfy/ lie in the margin of error, i.e. confidence level * Standard Error. Standard error in this case is equal to the ratio of population standard deviation and square root of sample size.^[[1]](#footnote-1)^

We have considered a number of samples and checked for standard error in this sample on 95% confidence interval. Some samples satisfied the evaluation criteria, and some didn’t. We have selected one of the randomly selected sample, whose mean was within the range of margin of error. Population mean, sample mean for selected sample, standard error and the confidence interval are shown in table A_2_.

Table A_2_:

| Population Mean | 0.7695 |
| --- | --- |
| population standard deviation | 1.588968 |
| Standard Error | 0.001285 |
| SE*95%confidence interval | 0.011242 |
| Margin of error | (0.75667,0.77915) |
| Sample Mean | 0.76791 |

1. D.M.Deiaz, C.D. Barr and M. Cetinkaya-Rundel. *OpenIntro Statistics*. Create Space, 2015, p. 173. [↑](#footnote-ref-1)
