# Supplementary material for: Comparison of researchers’ impact indices
Source: PLoS One. 2020 May 29;15(5):e0233765. doi: 10.1371/journal.pone.0233765 (PMC7259586; doi:10.1371/journal.pone.0233765)
Supplement: S2 Appendix — (DOCX) [file pone.0233765.s002.docx]

Appendix B

Table A_3_: Societies and Number of Awardees

| Societies and their Awards | Total Awardees |
| --- | --- |
| ACM |  |
| ACM Turing Award | 60 |
| ACM Fellow | 958 |
| software system award | 120 |
| Gordon Bell Prize | 161 |
| Karl V. Karlstrom Outstanding educator Award | 24 |
| Outstanding Contribution to ACM Award | 45 |
| Doctoral Dissertation Award | 92 |
| Grace Murray Hopper Award | 40 |
| Paris Kanellakis Theory and Practice award | 40 |
| Distinguished Service Award | 44 |
| Infosys Foundation Award | 8 |
| Programming Languages Achievement Award | 19 |
| Eugene L. Lawler Award | 10 |
| AAAI Allen Newell Award | 21 |
| ACM Presidential Award | 17 |
| ACM prize in comp. science and Engg. | 14 |
| Programming system and language award | 31 |
| ACM-W Athena Lecture Award | 9 |
| ACM-IEEE CS Eckert-Mauchly Award | 36 |
| ACM-IEEE CS George Michael HPC Fellowships | 31 |
| Total | 1780 |
| IEEE |  |
| IEEE Seymour Cray Award | 16 |
| IEEE Computer Pioneer Award | 94 |
| IEEE technical achievement award | 75 |
| IEEE John von Neumann Medal | 27 |
| Total | 212 |
| Grand Total | 1992 |
