# Supplementary material for: Comparison of researchers’ impact indices
Source: PLoS One. 2020 May 29;15(5):e0233765. doi: 10.1371/journal.pone.0233765 (PMC7259586; doi:10.1371/journal.pone.0233765)
Supplement: S3 Appendix — (DOCX) [file pone.0233765.s003.docx]

Appendix C**:** List of Award winners

| **ACM TURING AWARD** | | | | | | | |
| --- | --- | --- | --- | --- | --- | --- | --- |
| **Sr. #** | **First name** | **Last Name** | **year** | **Sr. #** | **First name** | **Last Name** | **year** |
| 1 | Alan J. | Perlis | 1966 | 37 | Amir | Pnueli | 1996 |
| 2 | Maurice V. | Wilkes | 1967 | 38 | Douglas | Engelbart | 1997 |
| 3 | Richard W. | Hamming | 1968 | 39 | Jim | Gray | 1998 |
| 4 | Marvin L. | Minsky | 1969 | 40 | Frederick P. | Brooks | 1999 |
| 5 | James H. | Wilkinson | 1970 | 41 | Andrew Chi-Chih | Yao | 2000 |
| 6 | John | McCarthy | 1971 | 42 | Ole-Johan | Dahl | 2001 |
| 7 | Edsger W. | Dijkstra | 1972 | 43 | Kristen | Nygaard | 2001 |
| 8 | Charles W. | Bachman | 1973 | 44 | Leonard M. | Adleman | 2002 |
| 9 | Donald E. | Knuth | 1974 | 45 | Ronald L. | Rivest | 2002 |
| 10 | Allen | Newell | 1975 | 46 | Adi | Shamir | 2002 |
| 11 | Herbert A. | Simon | 1975 | 47 | Alan | Kay | 2003 |
| 12 | Michael O. | Rabin | 1976 | 48 | Vinton | Cerf | 2004 |
| 13 | Dana S. | Scott | 1976 | 49 | Robert E. | Kahn | 2004 |
| 14 | John | Backus | 1977 | 50 | Peter | Naur | 2005 |
| 15 | Robert W. | Floyd | 1978 | 51 | Frances E. | Allen | 2006 |
| 16 | Kenneth E. | Iverson | 1979 | 52 | Edmund M. | Clarke | 2007 |
| 17 | C. Antony R. | Hoare | 1980 | 53 | E. Allen | Emerson | 2007 |
| 18 | Edgar F. | Codd | 1981 | 54 | Joseph | Sifakis | 2007 |
| 19 | Stephen A. | Cook | 1982 | 55 | Barbara J.H. | Liskov | 2008 |
| 20 | Dennis M. | Ritchie | 1983 | 56 | Charles P. | Thacker | 2009 |
| 21 | Kenneth L. | Thompson | 1983 | 57 | Leslie | Valiant | 2010 |
| 22 | Niklaus E. | Wirth | 1984 | 58 | Judea | Pearl | 2011 |
| 23 | Richard M. | Karp | 1985 | 59 | ShafiMicali, Silvio | Goldwasser | 2012 |
| 24 | John E. | Hopcroft | 1986 | 60 | Leslie | Lamport | 2013 |
| 25 | Robert E. | Tarjan | 1986 |  |  |  |  |
| 26 | John | Cocke | 1987 |  |  |  |  |
| 27 | Ivan | Sutherland | 1988 |  |  |  |  |
| 28 | William | Kahan | 1989 |  |  |  |  |
| 29 | Fernando J. | Corbato | 1990 |  |  |  |  |
| 30 | A.J. Robin | Milner | 1991 |  |  |  |  |
| 31 | Butler W. | Lampson | 1992 |  |  |  |  |
| 32 | Juris | Hartmanis | 1993 |  |  |  |  |
| 33 | Richard E. | Stearns | 1993 |  |  |  |  |
| 34 | Edward | Feigenbaum | 1994 |  |  |  |  |
| 35 | Raj | Reddy | 1994 |  |  |  |  |
| 36 | Manuel | Blum | 1995 |  |  |  |  |

| **ACM FELLOW AWARD** | | | | | | | | | | | | | |  |
| --- | --- | --- | --- | --- | --- | --- | --- | --- | --- | --- | --- | --- | --- | --- |
| **Sr. #** | **First name** | **Last Name** | | | **year** | **Sr. #** | | **First name** | | **Last Name** | | **year** | |  |
| 61 | Abramsky | Samson | | | 2014 | 101 | | Shieber | | Stuart | | 2014 | |  |
| 62 | Adve | Vikram | | | 2014 | 102 | | Srikant | | Ramakrishnan | | 2014 | |  |
| 63 | Afrati | Foto | | | 2014 | 103 | | Srinivasan | | Aravind | | 2014 | |  |
| 64 | W Bachman | Charles | | | 2014 | 104 | | Sudarshan | | S. | | 2014 | |  |
| 65 | Borodin | Allan | | | 2014 | 105 | | Syverson | | Paul | | 2014 | |  |
| 66 | Bundy | Alan | | | 2014 | 106 | | Tsudik | | Gene | | 2014 | |  |
| 67 | Faith Cranor | Lorrie | | | 2014 | 107 | | Whittaker | | Steve | | 2014 | |  |
| 68 | A Davis | Timothy | | | 2014 | 108 | | S Ackerman | | Mark | | 2013 | |  |
| 69 | Devadas | Srinivas | | | 2014 | 109 | | C. Aggarwal | | Charu | | 2013 | |  |
| 70 | Dhillon | Inderjit | | | 2014 | 110 | | H Anderson | | James | | 2013 | |  |
| 71 | D. Dutt | Nikil | | | 2014 | 111 | | Bellare | | Mihir | | 2013 | |  |
| 72 | Ellen | Faith | | | 2014 | 112 | | L Borgman | | Christine | | 2013 | |  |
| 73 | D Ernst | Michael | | | 2014 | 113 | | Ceri | | Stefano | | 2013 | |  |
| 74 | Finkelstein | Adam | | | 2014 | 114 | | Chakrabarty | | Krishnendu | | 2013 | |  |
| 75 | Freire | Juliana | | | 2014 | 115 | | Chellappa | | Ramalingam | | 2013 | |  |
| 76 | Gehrke | Johannes | | | 2014 | 116 | | J. Cox | | Ingemar | | 2013 | |  |
| 77 | Grimson | Eric | | | 2014 | 117 | | J. P. De Lucena | | Carlos | | 2013 | |  |
| 78 | Guzdial | Mark | | | 2014 | 118 | | Dechter | | Rina | | 2013 | |  |
| 79 | Heiser | Gernot | | | 2014 | 119 | | Elliott | | Chip | | 2013 | |  |
| 80 | Horvitz | Eric | | | 2014 | 120 | | Forsyth | | David | | 2013 | |  |
| 81 | Joachims | Thorsten | | | 2014 | 121 | | Gao | | Wen | | 2013 | |  |
| 82 | Kearns | Michael | | | 2014 | 122 | | Garlan | | David | | 2013 | |  |
| 83 | King | Valerie | | | 2014 | 123 | | Gosling | | James | | 2013 | |  |
| 84 | Kraus | Sarit | | | 2014 | 124 | | Haas | | Peter | | 2013 | |  |
| 85 | Lamport | Leslie | | | 2014 | 125 | | Hearst | | Marti | | 2013 | |  |
| 86 | Malik | Sharad | | | 2014 | 126 | | Jarke | | Matthias | | 2013 | |  |
| 87 | Mansour | Yishay | | | 2014 | 127 | | K. Kannan | | Sampath | | 2013 | |  |
| 88 | Mitra | Subhasish | | | 2014 | 128 | | J Kasik | | David | | 2013 | |  |
| 89 | Mitzenmacher | Michael | | | 2014 | 129 | | Katabi | | Dina | | 2013 | |  |
| 90 | Morris | Robert | | | 2014 | 130 | | A Kautz | | Henry | | 2013 | |  |
| 91 | Narayanan | Vijaykrishnan | | | 2014 | 131 | | Kleinberg | | Jon | | 2013 | |  |
| 92 | B Navathe | Shamkant | | | 2014 | 132 | | Kumar | | Panganamala | | 2013 | |  |
| 93 | M Patel | Jignesh | | | 2014 | 133 | | S Lea | | Douglas | | 2013 | |  |
| 94 | Ranganathan | Parthasarathy | | | 2014 | 134 | | Maarek | | Yoelle | | 2013 | |  |
| 95 | Reingold | Omer | | | 2014 | 135 | | Manning | | Christopher | | 2013 | |  |
| 96 | Rodden | Tom | | | 2014 | 136 | | Marathe | | Madhav | | 2013 | |  |
| 97 | Rubinfeld | Ronitt | | | 2014 | 137 | | Mellor-Crummey | | John | | 2013 | |  |
| 98 | Rus | Daniela | | | 2014 | 138 | | Morrisett | | Greg | | 2013 | |  |
| 99 | Luigi Sangiovanni | Alberto | | | 2014 | 139 | | C. Myers | | Andrew | | 2013 | |  |
| 100 | Schulzrinne | Henning | | | 2014 | 140 | | Nau | | Dana | | 2013 | |  |
| **Sr. #** | **First name** | **Last Name** | | | **year** | **Sr. #** | | **First name** | | **Last Name** | | **year** | |  |
| 141 | Rao | Satish | | | 2013 | 186 | | O'Rourke | | Joseph | | 2012 | |  |
| 142 | E Robertson | S | | | 2013 | 187 | | C. Pierce | | Benjamin | | 2012 | |  |
| 143 | Roscoe | Timothy | | | 2013 | 188 | | K Pingali | | Keshav | | 2012 | |  |
| 144 | K Sellis | Timoleon | | | 2013 | 189 | | M Pitts | | Andrew | | 2012 | |  |
| 145 | E Shasha | Dennis | | | 2013 | 190 | | R. Rastogi | | Rajeev | | 2012 | |  |
| 146 | N Shavit | Nir | | | 2013 | 191 | | Reddy | | Raj | | 2012 | |  |
| 147 | Shim | Kyuseok | | | 2013 | 192 | | Ross | | Keith | | 2012 | |  |
| 148 | Smyth | Padhraic | | | 2013 | 193 | | Sakallah | | Karem | | 2012 | |  |
| 149 | Tambe | Milind | | | 2013 | 194 | | S Schreiber | | Robert | | 2012 | |  |
| 150 | Tannen | Val | | | 2013 | 195 | | Scott | | Steven | | 2012 | |  |
| 151 | P. Williamson | David | | | 2013 | 196 | | Selman | | Bart | | 2012 | |  |
| 152 | Wong | Limsoon | | | 2013 | 197 | | Shamir | | Ron | | 2012 | |  |
| 153 | Yung | Moti | | | 2013 | 198 | | Shoham | | Yoav | | 2012 | |  |
| 154 | Zegura | Ellen | | | 2013 | 199 | | Sifakis | | Joseph | | 2012 | |  |
| 155 | Zhang | Zhengyou | | | 2013 | 200 | | Sinclair | | Alistair | | 2012 | |  |
| 156 | Zhou | Yuanyuan | | | 2013 | 201 | | Stein | | Clifford | | 2012 | |  |
| 157 | Zuckerman | David | | | 2013 | 202 | | Stoica | | Ion | | 2012 | |  |
| 158 | Alonso | Gustavo | | | 2012 | 203 | | Tamassia | | Roberto | | 2012 | |  |
| 159 | Arge | Lars | | | 2012 | 204 | | F. Tichy | | Walter | | 2012 | |  |
| 160 | Baldi | Pierre | | | 2012 | 205 | | Valduriez | | Patrick | | 2012 | |  |
| 161 | Boehm | Hans-J. | | | 2012 | 206 | | G Valiant | | Leslie | | 2012 | |  |
| 162 | Boutilier | Craig | | | 2012 | 207 | | Yelick | | Kathy | | 2012 | |  |
| 163 | K Camp | Tracy | | | 2012 | 208 | | Zabih | | Ramin | | 2012 | |  |
| 164 | Cattell | Rick | | | 2012 | 209 | | Zhang | | Xiaodong | | 2012 | |  |
| 165 | S. Davis | Larry | | | 2012 | 210 | | Abiteboul | | Serge | | 2011 | |  |
| 166 | Elmagarmid | Ahmed | | | 2012 | 211 | | Agrawal | | Divyakant | | 2011 | |  |
| 167 | Fan | Wenfei | | | 2012 | 212 | | M. Baecker | | Ronald | | 2011 | |  |
| 168 | Gao | Lixin | | | 2012 | 213 | | J. Ball | | Thomas | | 2011 | |  |
| 169 | Garfinkel | Simson | | | 2012 | 214 | | Blelloch | | Guy | | 2011 | |  |
| 170 | A. Gibson | Garth | | | 2012 | 215 | | Ebeling | | Carl | | 2011 | |  |
| 171 | Greenberg | Saul | | | 2012 | 216 | | Eppstein | | David | | 2011 | |  |
| 172 | Gross | Markus | | | 2012 | 217 | | Fox | | Geoffrey | | 2011 | |  |
| 173 | P. Grove | David | | | 2012 | 218 | | Furnas | | George | | 2011 | |  |
| 174 | Grudin | Jonathan | | | 2012 | 219 | | K. Gifford | | David | | 2011 | |  |
| 175 | Guerraoui | Rachid | | | 2012 | 220 | | Govindan | | Ramesh | | 2011 | |  |
| 176 | Gupta | Manish | | | 2012 | 221 | | Guo | | Baining | | 2011 | |  |
| 177 | Hershberger | John | | | 2012 | 222 | | Heckerman | | David | | 2011 | |  |
| 178 | Kahng | Andrew | | | 2012 | 223 | | J. Holzmann | | Gerard | | 2011 | |  |
| 179 | Karlin | Anna | | | 2012 | 224 | | Hoppe | | Hugues | | 2011 | |  |
| 180 | Keshav | Srinivasan | | | 2012 | 225 | | S. Jensen | | Christian | | 2011 | |  |
| 181 | Kiczales | Gregor | | | 2012 | 226 | | J. Karloff | | Howard | | 2011 | |  |
| 182 | Kitsuregawa | Masaru | | | 2012 | 227 | | Keckler | | Stephen | | 2011 | |  |
| 183 | Libkin | Leonid | | | 2012 | 228 | | B. Key | | Peter | | 2011 | |  |
| 184 | Milo | Tova | | | 2012 | 229 | | Kirkpatrick | | Scott | | 2011 | |  |
| 185 | Nahrstedt | Klara | | | 2012 | 230 | | Kraut | | Robert | | 2011 | |  |
| **Sr. #** | **First name** | **Last Name** | | | **year** | **Sr. #** | | **First name** | | **Last Name** | | **year** | |  |
| 231 | Landau | Susan | | | 2011 | 276 | | Kiesler | | Sara | | 2010 | |  |
| 232 | C. Lin | Ming | | | 2011 | 277 | | N. Klein | | Philip | | 2010 | |  |
| 233 | S. Magnusson | Peter | | | 2011 | 278 | | Kossmann | | Donald | | 2010 | |  |
| 234 | Malkhi | Dahlia | | | 2011 | 279 | | Launchbury | | John | | 2010 | |  |
| 235 | Marzullo | Keith | | | 2011 | 280 | | F. Lyon | | Richard | | 2010 | |  |
| 236 | Matsuoka | Satoshi | | | 2011 | 281 | | Mooney | | Raymond | | 2010 | |  |
| 237 | Max | Nelson | | | 2011 | 282 | | Muthukrishnan | | S. | | 2010 | |  |
| 238 | Mitchell | Joseph | | | 2011 | 283 | | Pereira | | Fernando | | 2010 | |  |
| 239 | Mukherjee | Shubu | | | 2011 | 284 | | Pevzner | | Pavel | | 2010 | |  |
| 240 | Chin Ooi | Beng | | | 2011 | 285 | | Rombach | | Dieter | | 2010 | |  |
| 241 | Ozsoyoglu | Zehra | | | 2011 | 286 | | S. Rosenblum | | David | | 2010 | |  |
| 242 | Pach | Janos | | | 2011 | 287 | | Savage | | Stefan | | 2010 | |  |
| 243 | Petzold | Linda | | | 2011 | 288 | | B. Schnabel | | Robert | | 2010 | |  |
| 244 | Pollack | Martha | | | 2011 | 289 | | A. Spielman | | Daniel | | 2010 | |  |
| 245 | Roth | Dan | | | 2011 | 290 | | Suri | | Subhash | | 2010 | |  |
| 246 | Sanguinetti | John | | | 2011 | 291 | | Wm. Tompa | | Frank | | 2010 | |  |
| 247 | Seltzer | Margo | | | 2011 | 292 | | Torrellas | | Josep | | 2010 | |  |
| 248 | Singhal | Amit | | | 2011 | 293 | | Trimberger | | Stephen | | 2010 | |  |
| 249 | L. Souvaine | Diane | | | 2011 | 294 | | M. Ungar | | David | | 2010 | |  |
| 250 | Srivastava | Divesh | | | 2011 | 295 | | Zeller | | Andreas | | 2010 | |  |
| 251 | Suciu | Dan | | | 2011 | 296 | | Zhai | | Shumin | | 2010 | |  |
| 252 | M. Tullsen | Dean | | | 2011 | 297 | | Attiya | | Hagit | | 2009 | |  |
| 253 | Vahdat | Amin | | | 2011 | 298 | | F. Bacon | | David | | 2009 | |  |
| 254 | Wetherall | David | | | 2011 | 299 | | A. Baeza-Yates | | Ricardo | | 2009 | |  |
| 255 | Kenneth Zadeck | Frank | | | 2011 | 300 | | L. Bajaj | | Chandrajit | | 2009 | |  |
| 256 | A Abramson | David | | | 2010 | 301 | | P Bhatkar | | Vijay | | 2009 | |  |
| 257 | Adve | Sarita | | | 2010 | 302 | | A. Blakeley | | Jose | | 2009 | |  |
| 258 | Alvisi | Lorenzo | | | 2010 | 303 | | Borriello | | Gaetano | | 2009 | |  |
| 259 | Andre Barroso | Luiz | | | 2010 | 304 | | Choudhary | | Alok | | 2009 | |  |
| 260 | C Burger | Doug | | | 2010 | 305 | | B. Dale | | Nell | | 2009 | |  |
| 261 | Chayes | Jennifer | | | 2010 | 306 | | Davie | | Bruce | | 2009 | |  |
| 262 | Chen | Peter | | | 2010 | 307 | | A. Dean | | Jeffrey | | 2009 | |  |
| 263 | Condon | Anne | | | 2010 | 308 | | L. Dean | | Thomas | | 2009 | |  |
| 264 | Crovella | Mark | | | 2010 | 309 | | R. Donald | | Bruce | | 2009 | |  |
| 265 | Cytron | Ron | | | 2010 | 310 | | D Erickson | | Thomas | | 2009 | |  |
| 266 | D. Dahlin | Michael | | | 2010 | 311 | | Fischer | | Gerhard | | 2009 | |  |
| 267 | El El Abbadi | Amr | | | 2010 | 312 | | T. Foster | | Ian | | 2009 | |  |
| 268 | S. Ellis | Carla | | | 2010 | 313 | | V. Goldberg | | Andrew | | 2009 | |  |
| 269 | Faloutsos | Christos | | | 2010 | 314 | | T. Goodrich | | Michael | | 2009 | |  |
| 270 | S. Fisher | Kathleen | | | 2010 | 315 | | Govindaraju | | Venugopal | | 2009 | |  |
| 271 | Goodman | James | | | 2010 | 316 | | Gupta | | Rajiv | | 2009 | |  |
| 272 | Hall | Wendy | | | 2010 | 317 | | M Hellerstein | | Joseph | | 2009 | |  |
| 273 | Hubaux | Jean-Pierre | | | 2010 | 318 | | J Hendren | | Laurie | | 2009 | |  |
| 274 | I. Jordan | Michael | | | 2010 | 319 | | Hoelzle | | Urs | | 2009 | |  |
| 275 | Kavraki | Lydia | | | 2010 | 320 | | Jahanian | | Farnam | | 2009 | |  |
| **Sr. #** | **First name** | **Last Name** | | | **year** | **Sr. #** | | **First name** | | **Last Name** | | **year** | |  |
| 321 | L. Kaltofen | Erich | | | 2009 | 363 | | A. Konstan | | Joseph | | 2008 | |  |
| 322 | Karger | David | | | 2009 | 364 | | Levin | | Roy | | 2008 | |  |
| 323 | E. Kaufman | Arie | | | 2009 | 365 | | Geoffrey Lowney | | P. | | 2008 | |  |
| 324 | Kriegel | Hans-Peter | | | 2009 | 366 | | Malik | | Jitendra | | 2008 | |  |
| 325 | Lenzerini | Maurizio | | | 2009 | 367 | | Mckinley | | Kathryn | | 2008 | |  |
| 326 | C.S. Lui | John | | | 2009 | 368 | | Meyer | | Bertrand | | 2008 | |  |
| 327 | Manocha | Dinesh | | | 2009 | 369 | | C. Mitchell | | John | | 2008 | |  |
| 328 | Martonosi | Margaret | | | 2009 | 370 | | Moses | | Joel | | 2008 | |  |
| 329 | Matias | Yossi | | | 2009 | 371 | | Munro | | J. | | 2008 | |  |
| 330 | J. Miller | Renee | | | 2009 | 372 | | S. Olson | | Judith | | 2008 | |  |
| 331 | T. Riedl | John | | | 2009 | 373 | | C. Paulson | | Lawrence | | 2008 | |  |
| 332 | Rinard | Martin | | | 2009 | 374 | | Pirahesh | | Hamid | | 2008 | |  |
| 333 | G. Selinger | Patricia | | | 2009 | 375 | | Randell | | Brian | | 2008 | |  |
| 334 | Kallikote Shyamasundar | Rudrapatna | | | 2009 | 376 | | Reiter | | Michael | | 2008 | |  |
| 335 | Teng | Shang-Hua | | | 2009 | 377 | | Rexford | | Jennifer | | 2008 | |  |
| 336 | A. Thekkath | Chandramohan | | | 2009 | 378 | | Rose | | Jonathan | | 2008 | |  |
| 337 | Van Renesse | Robbert | | | 2009 | 379 | | Rosenblum | | Mendel | | 2008 | |  |
| 338 | C. Vemuri | Baba | | | 2009 | 380 | | A. Rutenbar | | Rob | | 2008 | |  |
| 339 | J Verissimo | Paulo | | | 2009 | 381 | | Sandholm | | Tuomas | | 2008 | |  |
| 340 | Vetterli | Martin | | | 2009 | 382 | | Sarkar | | Vivek | | 2008 | |  |
| 341 | Whang | Kyu-Young | | | 2009 | 383 | | S. Squillante | | Mark | | 2008 | |  |
| 342 | Wilks | Yorick | | | 2009 | 384 | | O Stenstrom | | Per | | 2008 | |  |
| 343 | Winograd | Terry | | | 2009 | 385 | | Sudan | | Madhu | | 2008 | |  |
| 344 | Abadi | Martin | | | 2008 | 386 | | Szeliski | | Richard | | 2008 | |  |
| 345 | Abowd | Gregory | | | 2008 | 387 | | B Terry | | Douglas | | 2008 | |  |
| 346 | Aiken | Alexander | | | 2008 | 388 | | Agarwal | | Anant | | 2007 | |  |
| 347 | Arora | Sanjeev | | | 2008 | 389 | | Alur | | Rajeev | | 2007 | |  |
| 348 | Balakrishnan | Hari | | | 2008 | 390 | | Banerjee | | Utpal | | 2007 | |  |
| 349 | A.S. Buxton | William | | | 2008 | 391 | | Beeri | | Catriel | | 2007 | |  |
| 350 | Clarkson | Kenneth | | | 2008 | 392 | | Blum | | Avrim | | 2007 | |  |
| 351 | Cong | Jason | | | 2008 | 393 | | A. Brewer | | Eric | | 2007 | |  |
| 352 | Cook | Perry | | | 2008 | 394 | | Broder | | Andrei | | 2007 | |  |
| 353 | A. Cook | Stephen | | | 2008 | 395 | | F. Cohen | | Michael | | 2007 | |  |
| 354 | Davidson | Jack | | | 2008 | 396 | | Constantine | | Larry | | 2007 | |  |
| 355 | Dayal | Umeshwar | | | 2008 | 397 | | Dolev | | Danny | | 2007 | |  |
| 356 | Deng | Xiaotie | | | 2008 | 398 | | Downey | | Rodney | | 2007 | |  |
| 357 | Garcia-Luna-Aceves | Jose | | | 2008 | 399 | | A Feigenbaum | | Edward | | 2007 | |  |
| 358 | X Goemans | Michel | | | 2008 | 400 | | W. Felten | | Edward | | 2007 | |  |
| 359 | Hanrahan | Patrick | | | 2008 | 401 | | Fortnow | | Lance | | 2007 | |  |
| 360 | H. House | Charles | | | 2008 | 402 | | Gao | | Guang | | 2007 | |  |
| 361 | S. Humphrey | Watts | | | 2008 | 403 | | Gottlob | | Georg | | 2007 | |  |
| 362 | Kay | Alan | | | 2008 | 404 | | Hull | | Richard | | 2007 | |  |
| **Sr. #** | **First name** | **Last Name** | | | **year** | **Sr. #** | | **First name** | | **Last Name** | | **year** | |  |
| 405 | Huttenlocher | Daniel | | | 2007 | 450 | | Strother Moore | | J | | 2006 | |  |
| 406 | Jiang | Tao | | | 2007 | 451 | | Newell | | Alan | | 2006 | |  |
| 407 | C. Klensin | John | | | 2007 | 452 | | Norvig | | Peter | | 2006 | |  |
| 408 | S. Lam | Monica | | | 2007 | 453 | | P. O'Leary | | Dianne | | 2006 | |  |
| 409 | Levoy | Marc | | | 2007 | 454 | | R Olsen | | Dan | | 2006 | |  |
| 410 | Mishra | Bhubaneswar | | | 2007 | 455 | | A Olukotun | | Kunle | | 2006 | |  |
| 411 | Eliot Moss | J. | | | 2007 | 456 | | Ozsu | | Tamer | | 2006 | |  |
| 412 | Motwani | Rajeev | | | 2007 | 457 | | Paxson | | Vern | | 2006 | |  |
| 413 | M Odersky | Martin | | | 2007 | 458 | | Scott | | Michael | | 2006 | |  |
| 414 | M. Olson | Gary | | | 2007 | 459 | | Shum | | Harry | | 2006 | |  |
| 415 | Padua | David | | | 2007 | 460 | | Z Spector | | Alfred | | 2006 | |  |
| 416 | Pausch | Randy | | | 2007 | 461 | | Vianu | | Victor | | 2006 | |  |
| 417 | Pnueli | Amir | | | 2007 | 462 | | Winslett | | Marianne | | 2006 | |  |
| 418 | Prasanna | Viktor | | | 2007 | 463 | | L Wolf | | Alexander | | 2006 | |  |
| 419 | A. G. Requicha | Aristides | | | 2007 | 464 | | W York | | Bryant | | 2006 | |  |
| 420 | S. Roberts | Eric | | | 2007 | 465 | | Zdonik | | Stanley | | 2006 | |  |
| 421 | Terzopoulos | Demetri | | | 2007 | 466 | | Zhang | | Lixia | | 2006 | |  |
| 422 | E. Thomas | Donald | | | 2007 | 467 | | Anderson | | Thomas | | 2005 | |  |
| 423 | Wadler | Philip | | | 2007 | 468 | | Bjorner | | Dines | | 2005 | |  |
| 424 | Wand | Mitchell | | | 2007 | 469 | | Bourne | | Stephen | | 2005 | |  |
| 425 | Zhang | Hongjiang | | | 2007 | 470 | | A Brooks | | Rodney | | 2005 | |  |
| 426 | Allender | Eric | | | 2006 | 471 | | Chaudhuri | | Surajit | | 2005 | |  |
| 427 | Arvind |  | | | 2006 | 472 | | D Cooper | | Keith | | 2005 | |  |
| 428 | Atallah | Mikhail | | | 2006 | 473 | | Dill | | David | | 2005 | |  |
| 429 | Chen | Ming-Syan | | | 2006 | 474 | | Diot | | Christophe | | 2005 | |  |
| 430 | T Dumais | Susan | | | 2006 | 475 | | Dubois | | Michel | | 2005 | |  |
| 431 | Fayyad | Usama | | | 2006 | 476 | | J Franklin | | Michael | | 2005 | |  |
| 432 | Felleisen | Matthias | | | 2006 | 477 | | Frieder | | Ophir | | 2005 | |  |
| 433 | Forbus | Kenneth | | | 2006 | 478 | | Harper | | Robert | | 2005 | |  |
| 434 | B Gibbons | Phillip | | | 2006 | 479 | | Herlihy | | Maurice | | 2005 | |  |
| 435 | Lee Giles | C | | | 2006 | 480 | | G Kolaitis | | Phokion | | 2005 | |  |
| 436 | G Greenberg | Albert | | | 2006 | 481 | | Kumar | | Vipin | | 2005 | |  |
| 437 | D Gropp | William | | | 2006 | 482 | | V Lakshman | | T | | 2005 | |  |
| 438 | Guerin | Roch | | | 2006 | 483 | | A Myers | | Brad | | 2005 | |  |
| 439 | Guttag | John | | | 2006 | 484 | | M Nicol | | David | | 2005 | |  |
| 440 | M Haas | Laura | | | 2006 | 485 | | Palem | | Krishna | | 2005 | |  |
| 441 | Yitzchak Halevy | Alon | | | 2006 | 486 | | Reps | | Thomas | | 2005 | |  |
| 442 | C Hearn | Anthony | | | 2006 | 487 | | Sha | | Lui | | 2005 | |  |
| 443 | A Henzinger | Thomas | | | 2006 | 488 | | Thorup | | Mikkel | | 2005 | |  |
| 444 | Jouppi | Norman | | | 2006 | 489 | | Upfal | | Eli | | 2005 | |  |
| 445 | E Laird | John | | | 2006 | 490 | | Vazirani | | Umesh | | 2005 | |  |
| 446 | Larus | James | | | 2006 | 491 | | Vazirani | | Vijay | | 2005 | |  |
| 447 | E Leiserson | Charles | | | 2006 | 492 | | Want | | Roy | | 2005 | |  |
| 448 | Li | Ming | | | 2006 | 493 | | Weikum | | Gerhard | | 2005 | |  |
| 449 | Mckeown | Nick | | | 2006 | 494 | | C. Weiser | | Uri | | 2005 | |  |
| **Sr. #** | **First name** | **Last Name** | | | **year** | **Sr. #** | | **First name** | | **Last Name** | | **year** | |  |
| 495 | Weld | Daniel | | | 2005 | 539 | | Kozen | | Dexter | | 2003 | |  |
| 496 | Wellman | Michael | | | 2005 | 540 | | Lin | | Yi-Bing | | 2003 | |  |
| 497 | Widom | Jennifer | | | 2005 | 541 | | Mckeown | | Kathleen | | 2003 | |  |
| 498 | Willinger | Walter | | | 2005 | 542 | | P Moran | | Thomas | | 2003 | |  |
| 499 | A Wood | David | | | 2005 | 543 | | Myers | | Eugene | | 2003 | |  |
| 500 | Zhang | Hui | | | 2005 | 544 | | Partridge | | Craig | | 2003 | |  |
| 501 | Bose | Bella | | | 2004 | 545 | | A Reed | | Daniel | | 2003 | |  |
| 502 | A Bubenko | Janis | | | 2004 | 546 | | J Russell | | Stuart | | 2003 | |  |
| 503 | Cardelli | Luca | | | 2004 | 547 | | H Sanders | | William | | 2003 | |  |
| 504 | A Chien | Andrew | | | 2004 | 548 | | J Shenker | | Scott | | 2003 | |  |
| 505 | E Collins | George | | | 2004 | 549 | | S Sohi | | Gurindar | | 2003 | |  |
| 506 | Emer | Joel | | | 2004 | 550 | | J Van Rijsbergen | | C | | 2003 | |  |
| 507 | Gottlieb | Allan | | | 2004 | 551 | | Agarwal | | Pankaj | | 2002 | |  |
| 508 | Hanson | Vicki | | | 2004 | 552 | | D Agrawal | | Vishwani | | 2002 | |  |
| 509 | Hill | Mark | | | 2004 | 553 | | Babaoglu | | Ozalp | | 2002 | |  |
| 510 | E Ioannidis | Yannis | | | 2004 | 554 | | Crowcroft | | Jon | | 2002 | |  |
| 511 | Kaashoek | Frans | | | 2004 | 555 | | Culler | | David | | 2002 | |  |
| 512 | Larson | Per-Ake | | | 2004 | 556 | | J Dally | | William | | 2002 | |  |
| 513 | Lee | Peter | | | 2004 | 557 | | G Dietterich | | Thomas | | 2002 | |  |
| 514 | Mockapetris | Paul | | | 2004 | 558 | | Eggers | | Susan | | 2002 | |  |
| 515 | L Peyton-Jones | Simon | | | 2004 | 559 | | N Gabow | | Harold | | 2002 | |  |
| 516 | Schantz | Richard | | | 2004 | 560 | | Goyal | | Ambuj | | 2002 | |  |
| 517 | D Schroeder | Michael | | | 2004 | 561 | | Guzman | | Adolfo | | 2002 | |  |
| 518 | Vassiliadis | Stamatis | | | 2004 | 562 | | Halpern | | Joseph | | 2002 | |  |
| 519 | Wah | Benjamin | | | 2004 | 563 | | Hwu | | Wen-Mei | | 2002 | |  |
| 520 | S Wise | David | | | 2004 | 564 | | Immerman | | Neil | | 2002 | |  |
| 521 | Agrawal | Rakesh | | | 2003 | 565 | | Karin | | Sidney | | 2002 | |  |
| 522 | H Ammar | Mostafa | | | 2003 | 566 | | A Kellogg | | Wendy | | 2002 | |  |
| 523 | Bahl | Victor | | | 2003 | 567 | | B Lomet | | David | | 2002 | |  |
| 524 | Berger | Bonnie | | | 2003 | 568 | | L Miller | | Gary | | 2002 | |  |
| 525 | Bertino | Elisa | | | 2003 | 569 | | Mohan | | C. | | 2002 | |  |
| 526 | M Carroll | John | | | 2003 | 570 | | F Naughton | | Jeffrey | | 2002 | |  |
| 527 | Demillo | Richard | | | 2003 | 571 | | R Rau | | Bantwal | | 2002 | |  |
| 528 | J Grosz | Barbara | | | 2003 | 572 | | H Salesin | | David | | 2002 | |  |
| 529 | T Hailpern | Brent | | | 2003 | 573 | | Satyanarayanan | | Mahadev | | 2002 | |  |
| 530 | Han | Jiawei | | | 2003 | 574 | | Valero | | Mateo | | 2002 | |  |
| 531 | Harrold | Mary | | | 2003 | 575 | | Varghese | | George | | 2002 | |  |
| 532 | Hart | Peter | | | 2003 | 576 | | Wilkes | | John | | 2002 | |  |
| 533 | A Horowitz | Mark | | | 2003 | 577 | | A Abraham | | Jacob | | 2001 | |  |
| 534 | Hudak | Paul | | | 2003 | 578 | | M Aiken | | Robert | | 2001 | |  |
| 535 | V Jagadish | H | | | 2003 | 579 | | Asano | | Tetsuo | | 2001 | |  |
| 536 | K Jain | Anil | | | 2003 | 580 | | A Bernstein | | Philip | | 2001 | |  |
| 537 | C Jain | Ramesh | | | 2003 | 581 | | S Birnbaum | | Joel | | 2001 | |  |
| 538 | Jha | Niraj | | | 2003 | 582 | | H Borning | | Alan | | 2001 | |  |
| **Sr. #** | **First name** | **Last Name** | | | **year** | **Sr. #** | | **First name** | | **Last Name** | | **year** | |  |
| 583 | Breitbart | Yuri | | | 2001 | 627 | | G Shin | | Kang | | 2001 | |  |
| 584 | Cai | Jin-Yi | | | 2001 | 628 | | Shmoys | | David | | 2001 | |  |
| 585 | D Clark | David | | | 2001 | 629 | | Smith | | Alan | | 2001 | |  |
| 586 | B Davidson | Susan | | | 2001 | 630 | | Steinmetz | | Ralf | | 2001 | |  |
| 587 | Dekleer | Johan | | | 2001 | 631 | | Turner | | Jonathan | | 2001 | |  |
| 588 | Dongarra | Jack | | | 2001 | 632 | | Claire Wolf | | Marilyn | | 2001 | |  |
| 589 | J. Farber | David | | | 2001 | 633 | | Wolfson | | Ouri | | 2001 | |  |
| 590 | Feigenbaum | Joan | | | 2001 | 634 | | Zave | | Pamela | | 2001 | |  |
| 591 | Ferrari | Domenico | | | 2001 | 635 | | Banerjee | | Prithviraj | | 2000 | |  |
| 592 | J Floyd | Sally | | | 2001 | 636 | | Berman | | Francine | | 2000 | |  |
| 593 | Gelenbe | Erol | | | 2001 | 637 | | N Bhuyan | | Laxmi | | 2000 | |  |
| 594 | P Hayes | John | | | 2001 | 638 | | W Biermann | | Alan | | 2000 | |  |
| 595 | Iyengar | Sitharama | | | 2001 | 639 | | H Bokhari | | Shahid | | 2000 | |  |
| 596 | Iyer | Ravishankar | | | 2001 | 640 | | E Bryant | | Randal | | 2000 | |  |
| 597 | F Jaja | Joseph | | | 2001 | 641 | | Buneman | | Peter | | 2000 | |  |
| 598 | E Kahn | Robert | | | 2001 | 642 | | K Card | | Stuart | | 2000 | |  |
| 599 | Mo Kang | Sung | | | 2001 | 643 | | J Carey | | Michael | | 2000 | |  |
| 600 | B Kieburtz | Richard | | | 2001 | 644 | | E Comer | | Douglas | | 2000 | |  |
| 601 | A Kowalski | Robert | | | 2001 | 645 | | Duncan | | Karen | | 2000 | |  |
| 602 | Kramer | Jeffrey | | | 2001 | 646 | | Estrin | | Deborah | | 2000 | |  |
| 603 | Kurose | James | | | 2001 | 647 | | Fagin | | Ronald | | 2000 | |  |
| 604 | B Lee | Ruby | | | 2001 | 648 | | A Freeman | | Peter | | 2000 | |  |
| 605 | Litwin | Witold | | | 2001 | 649 | | Kent Fuchs | | W | | 2000 | |  |
| 606 | Micheli | Giovanni | | | 2001 | 650 | | J Haderle | | Donald | | 2000 | |  |
| 607 | P Miller | Barton | | | 2001 | 651 | | T Heath | | Michael | | 2000 | |  |
| 608 | C Mogul | Jeffrey | | | 2001 | 652 | | Kleinrock | | Leonard | | 2000 | |  |
| 609 | A. Norman | Donald | | | 2001 | 653 | | F Korth | | Henry | | 2000 | |  |
| 610 | M Pancake | Cherri | | | 2001 | 654 | | Van Lamsweerde | | Axel | | 2000 | |  |
| 611 | Papadimitriou | Christos | | | 2001 | 655 | | A Lorie | | Raymond | | 2000 | |  |
| 612 | B Parker | Donn | | | 2001 | 656 | | W Loveland | | Donald | | 2000 | |  |
| 613 | H Patel | Janak | | | 2001 | 657 | | R Meyer | | Albert | | 2000 | |  |
| 614 | Patt | Yale | | | 2001 | 658 | | H Morris | | James | | 2000 | |  |
| 615 | Pohl | Ira | | | 2001 | 659 | | L Peterson | | Larry | | 2000 | |  |
| 616 | Mark Pullen | J | | | 2001 | 660 | | Y Vardi | | Moshe | | 2000 | |  |
| 617 | Raghavan | Prabhakar | | | 2001 | 661 | | S Warren | | David | | 2000 | |  |
| 618 | Ramakrishnan | Raghu | | | 2001 | 662 | | Wilhelm | | Reinhard | | 2000 | |  |
| 619 | Ramamritham | Krithivasan | | | 2001 | 663 | | Williams | | Robin | | 2000 | |  |
| 620 | C Reynolds | John | | | 2001 | 664 | | E Zwaenepoel | | Willy | | 2000 | |  |
| 621 | G Robertson | George | | | 2001 | 665 | | Auslander | | Marc | | 1999 | |  |
| 622 | Roussopoulos | Nick | | | 2001 | 666 | | Birman | | Kenneth | | 1999 | |  |
| 623 | K. Sabnani | Krishnan | | | 2001 | 667 | | Brachman | | Ronald | | 1999 | |  |
| 624 | S Sandhu | Ravinderpal | | | 2001 | 668 | | T Braden | | Robert | | 1999 | |  |
| 625 | Schek | Hans-Joerg | | | 2001 | 669 | | Cook | | Robert | | 1999 | |  |
| 626 | D Schlichting | Richard | | | 2001 | 670 | | S Deblasi | | Joseph | | 1999 | |  |
| **Sr. #** | **First name** | **Last Name** | | | **year** | **Sr. #** | | **First name** | | **Last Name** | | **year** | |  |
| 671 | Demmel | James | | | 1999 | 716 | | T Kent | | Stephen | | 1998 | |  |
| 672 | J Fateman | Richard | | | 1999 | 717 | | Lam | | Simon | | 1998 | |  |
| 673 | D Foley | James | | | 1999 | 718 | | Li | | Kai | | 1998 | |  |
| 674 | D Gannon | John | | | 1999 | 719 | | Maier | | David | | 1998 | |  |
| 675 | M Geschke | C | | | 1999 | 720 | | S Notkin | | David | | 1998 | |  |
| 676 | Ghezzi | Carlo | | | 1999 | 721 | | H Nycum | | Susan | | 1998 | |  |
| 677 | L Glass | Robert | | | 1999 | 722 | | J Osterweil | | Leon | | 1998 | |  |
| 678 | L Graham | Ronald | | | 1999 | 723 | | P Rangan | | Venkat | | 1998 | |  |
| 679 | J Guibas | Leonidas | | | 1999 | 724 | | T Richards | | John | | 1998 | |  |
| 680 | Ibaraki | Toshihide | | | 1999 | 725 | | A Rowe | | Lawrence | | 1998 | |  |
| 681 | Kanade | Takeo | | | 1999 | 726 | | G Ryder | | Barbara | | 1998 | |  |
| 682 | M Lewis | Philip | | | 1999 | 727 | | Selman | | Alan | | 1998 | |  |
| 683 | B Macqueen | David | | | 1999 | 728 | | H Sequin | | Carlo | | 1998 | |  |
| 684 | Martin | Dianne | | | 1999 | 729 | | Siegel | | Howard | | 1998 | |  |
| 685 | M Masinter | Larry | | | 1999 | 730 | | H. Spafford | | Eugene | | 1998 | |  |
| 686 | Mehlhorn | Kurt | | | 1999 | 731 | | Tardos | | Eva | | 1998 | |  |
| 687 | L Mills | David | | | 1999 | 732 | | N Taylor | | Richard | | 1998 | |  |
| 688 | Pradhan | Dhiraj | | | 1999 | 733 | | J Turner | | Albert | | 1998 | |  |
| 689 | Sameh | Ahmed | | | 1999 | 734 | | Welzl | | Emmerich | | 1998 | |  |
| 690 | Samuelson | Pamela | | | 1999 | 735 | | M Wing | | Jeannette | | 1998 | |  |
| 691 | Snir | Marc | | | 1999 | 736 | | Yannakakis | | Mihalis | | 1998 | |  |
| 692 | T Snodgrass | Richard | | | 1999 | 737 | | Zweben | | Stuart | | 1998 | |  |
| 693 | Lou Soffa | Mary | | | 1999 | 738 | | F Akyildiz | | Ian | | 1997 | |  |
| 694 | Jen Tan | Chung | | | 1999 | 739 | | E Baer | | Jean-Loup | | 1997 | |  |
| 695 | Torii | Koji | | | 1999 | 740 | | Basili | | Victor | | 1997 | |  |
| 696 | L Waltz | David | | | 1999 | 741 | | R Bate | | Roger | | 1997 | |  |
| 697 | Warnock | John | | | 1999 | 742 | | W Boehm | | Barry | | 1997 | |  |
| 698 | Yonezawa | Akinori | | | 1999 | 743 | | Chlamtac | | Imrich | | 1997 | |  |
| 699 | P Agrawal | Dharma | | | 1998 | 744 | | D Couger | | J | | 1997 | |  |
| 700 | R Andrews | Gregory | | | 1998 | 745 | | Croft | | W | | 1997 | |  |
| 701 | W Appel | Andrew | | | 1998 | 746 | | B Davis | | Gordon | | 1997 | |  |
| 702 | Berghel | Hal | | | 1998 | 747 | | P Dobkin | | David | | 1997 | |  |
| 703 | C Browne | James | | | 1998 | 748 | | Freeman | | Herbert | | 1997 | |  |
| 704 | S Cartwright | Robert | | | 1998 | 749 | | Garcia-Molina | | Hector | | 1997 | |  |
| 705 | P Chen | Peter | | | 1998 | 750 | | Greif | | Irene | | 1997 | |  |
| 706 | Clarke | Edmund | | | 1998 | 751 | | Gurevich | | Yuri | | 1997 | |  |
| 707 | Clarke | Lori | | | 1998 | 752 | | L Hennessy | | John | | 1997 | |  |
| 708 | J Cole | Richard | | | 1998 | 753 | | Kedem | | Zvi | | 1997 | |  |
| 709 | A Ellis | Clarence | | | 1998 | 754 | | A Kemmerer | | Richard | | 1997 | |  |
| 710 | Gabriel | Richard | | | 1998 | 755 | | W Lawson | | H | | 1997 | |  |
| 711 | Krishna Gupta | Gopal | | | 1998 | 756 | | Lee | | Der-Tsai | | 1997 | |  |
| 712 | Jay Horning | James | | | 1998 | 757 | | Lipton | | Richard | | 1997 | |  |
| 713 | Jones | Neil | | | 1998 | 758 | | A Lynch | | Nancy | | 1997 | |  |
| 714 | K Joshi | Aravind | | | 1998 | 759 | | Menasce | | Daniel | | 1997 | |  |
| 715 | Kandel | Abraham | | | 1998 | 760 | | Miller | | Raymond | | 1997 | |  |
| **Sr. #** | **First name** | **Last Name** | | | **year** | **Sr. #** | | **First name** | | **Last Name** | | **year** | |  |
| 761 | Perrott | Ronald | | | 1997 | 806 | | Liskov | | Barbara | | 1996 | |  |
| 762 | Pippenger | Nicholas | | | 1997 | 807 | | R Muntz | | Richard | | 1996 | |  |
| 763 | R Pratt | Vaughan | | | 1997 | 808 | | E Nance | | Richard | | 1996 | |  |
| 764 | H Reif | John | | | 1997 | 809 | | Nishizeki | | Takao | | 1996 | |  |
| 765 | Reiter | Raymond | | | 1997 | 810 | | Preas | | Bryan | | 1996 | |  |
| 766 | Schneck | Paul | | | 1997 | 811 | | Rao | | Trn | | 1996 | |  |
| 767 | Sedgewick | Robert | | | 1997 | 812 | | M Reingold | | Edward | | 1996 | |  |
| 768 | C Sevcik | Kenneth | | | 1997 | 813 | | R Rice | | John | | 1996 | |  |
| 769 | Sharir | Micha | | | 1997 | 814 | | Rosenberg | | Arnold | | 1996 | |  |
| 770 | C Shaw | Alan | | | 1997 | 815 | | K Sahni | | Sartaj | | 1996 | |  |
| 771 | Shneiderman | Ben | | | 1997 | 816 | | Samet | | Hanan | | 1996 | |  |
| 772 | Steiglitz | Kenneth | | | 1997 | 817 | | E Savage | | John | | 1996 | |  |
| 773 | F Towsley | Donald | | | 1997 | 818 | | Sethi | | Ravi | | 1996 | |  |
| 774 | J Weyuker | Elaine | | | 1997 | 819 | | M Shaw | | Mary | | 1996 | |  |
| 775 | Widmayer | Peter | | | 1997 | 820 | | Silberschatz | | Abraham | | 1996 | |  |
| 776 | Wilensky | Robert | | | 1997 | 821 | | A Stankovic | | John | | 1996 | |  |
| 777 | S Yu | Philip | | | 1997 | 822 | | Stockmeyer | | Larry | | 1996 | |  |
| 778 | Zanella | Paolo | | | 1997 | 823 | | S Tanenbaum | | Andrew | | 1996 | |  |
| 779 | Richards Adrion | William | | | 1996 | 824 | | K Vernon | | Mary | | 1996 | |  |
| 780 | V Aho | A | | | 1996 | 825 | | Vishkin | | Uzi | | 1996 | |  |
| 781 | Ahuja | Narendra | | | 1996 | 826 | | S Vitter | | Jeffrey | | 1996 | |  |
| 782 | B Akeley | Kurt | | | 1996 | 827 | | I Wasserman | | Anthony | | 1996 | |  |
| 783 | R Bajcsy | Ruzena | | | 1996 | 828 | | Wegman | | Mark | | 1996 | |  |
| 784 | V Bochmann | Gregor | | | 1996 | 829 | | W Weingarten | | Fred | | 1996 | |  |
| 785 | Borg | Anita | | | 1996 | 830 | | Witten | | Ian | | 1996 | |  |
| 786 | Chandrasekaran | B | | | 1996 | 831 | | C Yovits | | Marshall | | 1996 | |  |
| 787 | Chazelle | Bernard | | | 1996 | 832 | | W Abrahams | | Paul | | 1995 | |  |
| 788 | Deo | Narsingh | | | 1996 | 833 | | L Ashenhurst | | R | | 1995 | |  |
| 789 | Dodd | George | | | 1996 | 834 | | H Barr | | Alan | | 1995 | |  |
| 790 | L. Encarnacao | Jose | | | 1996 | 835 | | Bernstein | | Lawrence | | 1995 | |  |
| 791 | Ferrante | Jeanne | | | 1996 | 836 | | Booch | | Grady | | 1995 | |  |
| 792 | J Fischer | Michael | | | 1996 | 837 | | H Brandin | | David | | 1995 | |  |
| 793 | J Frailey | Dennis | | | 1996 | 838 | | P Brent | | Richard | | 1995 | |  |
| 794 | M Graham | Robert | | | 1996 | 839 | | C Carpenter | | Loren | | 1995 | |  |
| 795 | A Harrison | Michael | | | 1996 | 840 | | Catmull | | Edwin | | 1995 | |  |
| 796 | Heidelberger | Philip | | | 1996 | 841 | | L Constable | | Robert | | 1995 | |  |
| 797 | Jane Irwin | Mary | | | 1996 | 842 | | E Denning | | Dorothy | | 1995 | |  |
| 798 | Jaffe | Jeffrey | | | 1996 | 843 | | Dewitt | | David | | 1995 | |  |
| 799 | Jain | Raj | | | 1996 | 844 | | E Druffel | | Larry | | 1995 | |  |
| 800 | K Jones | Anita | | | 1996 | 845 | | Engeler | | Erwin | | 1995 | |  |
| 801 | H Katz | Randy | | | 1996 | 846 | | Feldman | | Stuart | | 1995 | |  |
| 802 | Klawe | Maria | | | 1996 | 847 | | Fuchs | | Henry | | 1995 | |  |
| 803 | H Landweber | Lawrence | | | 1996 | 848 | | Galil | | Zvi | | 1995 | |  |
| 804 | E Lesk | Michael | | | 1996 | 849 | | R Garey | | Michael | | 1995 | |  |
| 805 | M Levy | Henry | | | 1996 | 850 | | Ginsberg | | Myron | | 1995 | |  |
| **Sr. #** | **First name** | **Last Name** | | | **year** | **Sr. #** | | **First name** | | **Last Name** | | **year** | |  |
| 851 | B Goodenough | John | | | 1995 | 896 | | Borman | | Lorraine | | 1994 | |  |
| 852 | Greenberg | Donald | | | 1995 | 897 | | L Bradshaw | | Charles | | 1994 | |  |
| 853 | R J Grosch | Herbert | | | 1995 | 898 | | S Bricklin | | Daniel | | 1994 | |  |
| 854 | Herzog | Bertram | | | 1995 | 899 | | Brooks, Jr | | Frederick | | 1994 | |  |
| 855 | J Highland | Harold | | | 1995 | 900 | | K Brotz | | Douglas | | 1994 | |  |
| 856 | J Hoffman | Lance | | | 1995 | 901 | | R Burton | | Richard | | 1994 | |  |
| 857 | Ibarra | Oscar | | | 1995 | 902 | | G Canning | | Richard | | 1994 | |  |
| 858 | S Johnson | David | | | 1995 | 903 | | Carlson | | Walter | | 1994 | |  |
| 859 | B Jones | Cliff | | | 1995 | 904 | | Cerf | | Vinton | | 1994 | |  |
| 860 | W Kennedy | Kenneth | | | 1995 | 905 | | Chamberlin | | Donald | | 1994 | |  |
| 861 | Kim | Won | | | 1995 | 906 | | F Codd | | Edgar | | 1994 | |  |
| 862 | Kosaraju | S | | | 1995 | 907 | | Coffman | | E | | 1994 | |  |
| 863 | E Ladner | Richard | | | 1995 | 908 | | J Corbato | | Fernando | | 1994 | |  |
| 864 | Lakshmivarahan | S | | | 1995 | 909 | | G Cragon | | Harvey | | 1994 | |  |
| 865 | Lazowska | Edward | | | 1995 | 910 | | A D'Auria | | Thomas | | 1994 | |  |
| 866 | Leveson | Nancy | | | 1995 | 911 | | Defanti | | Thomas | | 1994 | |  |
| 867 | Misra | Jay | | | 1995 | 912 | | J. Denning | | Peter | | 1994 | |  |
| 868 | Nievergelt | J | | | 1995 | 913 | | Dennis | | Jack | | 1994 | |  |
| 869 | Oettinger | Anthony | | | 1995 | 914 | | Peter Deutsch | | L | | 1994 | |  |
| 870 | P Preparata | Franco | | | 1995 | 915 | | W Dijkstra | | Edsger | | 1994 | |  |
| 871 | F Rada | Roy | | | 1995 | 916 | | Dunwell | | Stephen | | 1994 | |  |
| 872 | J Rosenkrantz | Daniel | | | 1995 | 917 | | Presper Eckert | | J | | 1994 | |  |
| 873 | Salton | Gerard | | | 1995 | 918 | | Elias | | Peter | | 1994 | |  |
| 874 | B Schneider | Fred | | | 1995 | 919 | | L Engel | | Gerald | | 1994 | |  |
| 875 | Snyder | Larry | | | 1995 | 920 | | H Esbin | | John | | 1994 | |  |
| 876 | Suzuki | Norihisa | | | 1995 | 921 | | O Evans | | Bob | | 1994 | |  |
| 877 | D Ullman | Jeffrey | | | 1995 | 922 | | Feng | | Tse-Yun | | 1994 | |  |
| 878 | S Wallace | Chris | | | 1995 | 923 | | Finerman | | Aaron | | 1994 | |  |
| 879 | Wegner | Peter | | | 1995 | 924 | | W. Floyd | | Robert | | 1994 | |  |
| 880 | R. White | John | | | 1995 | 925 | | J Flynn | | Michael | | 1994 | |  |
| 881 | Turner Whitted | J | | | 1995 | 926 | | M Frankston | | Robert | | 1994 | |  |
| 882 | Wiederhold | Gio | | | 1995 | 927 | | L Friedman | | Frank | | 1994 | |  |
| 883 | Wong | Chak-Kuen | | | 1995 | 928 | | A Galler | | Bernard | | 1994 | |  |
| 884 | C Yao | Andrew | | | 1995 | 929 | | W Gear | | Charles | | 1994 | |  |
| 885 | Young | Paul | | | 1995 | 930 | | Goldberg | | Adele | | 1994 | |  |
| 886 | M Adams | James | | | 1994 | 931 | | C. Gotlieb | | Calvin | | 1994 | |  |
| 887 | Allen | Frances | | | 1994 | 932 | | L Graham | | Susan | | 1994 | |  |
| 888 | L Alt | Franz | | | 1994 | 933 | | Gray | | Jim | | 1994 | |  |
| 889 | F. Atchison | William | | | 1994 | 934 | | Green | | Cordell | | 1994 | |  |
| 890 | H Austing | Richard | | | 1994 | 935 | | Gries | | David | | 1994 | |  |
| 891 | E Batcher | Kenneth | | | 1994 | 936 | | Hammer | | Carl | | 1994 | |  |
| 892 | Gordon Bell | C | | | 1994 | 937 | | W Hamming | | Richard | | 1994 | |  |
| 893 | W. Blasgen | Michael | | | 1994 | 938 | | Harel | | David | | 1994 | |  |
| 894 | Bobrow | Daniel | | | 1994 | 939 | | H Harris | | Fred | | 1994 | |  |
| 895 | R Boggs | David | | | 1994 | 940 | | Hartmanis | | Juris | | 1994 | |  |
| **Sr. #** | **First name** | **Last Name** | | | **year** | **Sr. #** | | **First name** | | **Last Name** | | **year** | |  |
| 941 | Daniel Hillis | Danny | | | 1994 | 986 | | B Simons | | Barbara | | 1994 | |  |
| 942 | E Hopcroft | John | | | 1994 | 987 | | Sloan | | Martha | | 1994 | |  |
| 943 | Hull | Tom | | | 1994 | 988 | | R Slutz | | Donald | | 1994 | |  |
| 944 | N Hume | J | | | 1994 | 989 | | J Smith | | Burton | | 1994 | |  |
| 945 | D Huskey | Harry | | | 1994 | 990 | | E Stearns | | Richard | | 1994 | |  |
| 946 | Kahan | William | | | 1994 | 991 | | B Steel | | Thomas | | 1994 | |  |
| 947 | M Kaplan | Ronald | | | 1994 | 992 | | L Steele | | Guy | | 1994 | |  |
| 948 | Karp | Richard | | | 1994 | 993 | | Stone | | Harold | | 1994 | |  |
| 949 | E Knuth | Donald | | | 1994 | 994 | | Stonebraker | | Michael | | 1994 | |  |
| 950 | J Kuck | David | | | 1994 | 995 | | Strecker | | William | | 1994 | |  |
| 951 | E Kurtz | Thomas | | | 1994 | 996 | | Stroustrup | | Bjarne | | 1994 | |  |
| 952 | Kurzweil | Ray | | | 1994 | 997 | | Suppes | | Patrick | | 1994 | |  |
| 953 | W Lampson | Butler | | | 1994 | 998 | | Sussman | | Gerald | | 1994 | |  |
| 954 | S Lavenberg | Stephen | | | 1994 | 999 | | Sutherland | | Ivan | | 1994 | |  |
| 955 | Lederberg | Joshua | | | 1994 | 1000 | | A Taft | | Edward | | 1994 | |  |
| 956 | A Lee | John | | | 1994 | 1001 | | E Tarjan | | Robert | | 1994 | |  |
| 957 | Lehman | Meir | | | 1994 | 1002 | | W. Taylor | | Robert | | 1994 | |  |
| 958 | Lindsay | Bruce | | | 1994 | 1003 | | P Thacker | | Charles | | 1994 | |  |
| 959 | Currie Little | Joyce | | | 1994 | 1004 | | Traiger | | Irv | | 1994 | |  |
| 960 | Liu | C | | | 1994 | 1005 | | Traub | | Joseph | | 1994 | |  |
| 961 | Stuart Lynn | M | | | 1994 | 1006 | | Tucker | | Allen | | 1994 | |  |
| 962 | Maisel | Herbert | | | 1994 | 1007 | | Van Dam | | Andries | | 1994 | |  |
| 963 | Manna | Zohar | | | 1994 | 1008 | | H Ware | | Willis | | 1994 | |  |
| 964 | Mc Carthy | John | | | 1994 | 1009 | | Wecker | | Stuart | | 1994 | |  |
| 965 | Mccluskey | Edward | | | 1994 | 1010 | | Wegbreit | | Ben | | 1994 | |  |
| 966 | D Mccracken | Daniel | | | 1994 | 1011 | | A Weiss | | Eric | | 1994 | |  |
| 967 | R Mcjones | Paul | | | 1994 | 1012 | | John Wheeler | | David | | 1994 | |  |
| 968 | J Milner | A | | | 1994 | 1013 | | V Wilkes | | Maurice | | 1994 | |  |
| 969 | Minker | Jack | | | 1994 | 1014 | | Winograd | | Shmuel | | 1994 | |  |
| 970 | M Needham | Roger | | | 1994 | 1015 | | E Wirth | | Niklaus | | 1994 | |  |
| 971 | G Neumann | Peter | | | 1994 | 1016 | | J Wolfson | | Seymour | | 1994 | |  |
| 972 | Newborn | Monroe | | | 1994 | 1017 | | A Wulf | | William | | 1994 | |  |
| 973 | K Ousterhout | John | | | 1994 | 1018 | | A Zadeh | | L | | 1994 | |  |
| 974 | S Owicki | Susan | | | 1994 |  | |  | |  | |  | |  |
| 975 | Lorge Parnas | David | | | 1994 |  | |  | |  | |  | |  |
| 976 | Patterson | David | | | 1994 |  | |  | |  | |  | |  |
| 977 | B Poucher | William | | | 1994 |  | |  | |  | |  | |  |
| 978 | Ralston | Anthony | | | 1994 |  | |  | |  | |  | |  |
| 979 | L Rivest | Ronald | | | 1994 |  | |  | |  | |  | |  |
| 980 | Rosenfeld | Azriel | | | 1994 |  | |  | |  | |  | |  |
| 981 | Rulifson | Jeff | | | 1994 |  | |  | |  | |  | |  |
| 982 | E Sammet | Jean | | | 1994 |  | |  | |  | |  | |  |
| 983 | S Scott | Dana | | | 1994 |  | |  | |  | |  | |  |
| 984 | Siewiorek | Daniel | | | 1994 |  | |  | |  | |  | |  |
| 985 | A Simon | Herbert | | | 1994 |  | |  | |  | |  | |  |
| **IEEE TECHNICAL ACHEIVEMENT AWARD** | | | | | | | | | | | | | | |
| **Sr. #** | **First name** | | **Last Name** | **year** | | | **Sr. #** | | **First name** | | **Last Name** | | **year** | |
| 1019 | Bowyer | | Kevin | 2014 | | | 1059 | | Rao | | Nageswara | | 2005 | |
| 1020 | Bruce Croft | | W. | 2014 | | | 1060 | | H. Spafford | | Eugene | | 2005 | |
| 1021 | Devadas | | Srinivas | 2014 | | | 1061 | | Ghosh | | Sumit | | 2004 | |
| 1022 | F. Felzenszwalb | | Pedro | 2014 | | | 1062 | | Han | | Jiawei | | 2004 | |
| 1023 | Y. Zomaya | | Albert | 2014 | | | 1063 | | Phoha | | Shashi | | 2004 | |
| 1024 | Camenisch | | Jan | 2013 | | | 1064 | | Sandhu | | Ravi | | 2004 | |
| 1025 | D. Gligor | | Virgil | 2013 | | | 1065 | | Chu | | Wesley | | 2003 | |
| 1026 | Tan | | Kian-Lee | 2013 | | | 1066 | | Jain | | Anil | | 2003 | |
| 1027 | Tardos | | Eva | 2013 | | | 1067 | | Kopetz | | Hermann | | 2003 | |
| 1028 | S. Yu | | Philip | 2013 | | | 1068 | | H. Sequin | | Carlo | | 2003 | |
| 1029 | Franz | | Michael | 2012 | | | 1069 | | Singhal | | Mukesh | | 2003 | |
| 1030 | Liu | | Ling | 2012 | | | 1070 | | Bertino | | Elisa | | 2002 | |
| 1031 | Nahrstedt | | Klara | 2012 | | | 1071 | | Chillarege | | Ram | | 2002 | |
| 1032 | Shyu | | Mei-Ling | 2012 | | | 1072 | | Ghafoor | | Arif | | 2000 | |
| 1033 | Wu | | Xindong | 2012 | | | 1073 | | M. Bolle | | Ruud | | 2000 | |
| 1034 | Gehrke | | Johannes | 2011 | | | 1074 | | Patnaik | | L.M. | | 1999 | |
| 1035 | Fagin | | Ronald | 2011 | | | 1075 | | Bhargava | | Bharat | | 1999 | |
| 1036 | Zhang | | Liang-Jie | 2011 | | | 1076 | | Chen | | Wen-Tsuen | | 1999 | |
| 1037 | Darema | | Frederica | 2011 | | | 1077 | | G. Bourbakis | | Nikolaos | | 1998 | |
| 1038 | Garcia-Luna-Aceves | | Jose | 2011 | | | 1078 | | Ichikawa | | Tadao | | 1998 | |
| 1039 | Grandison | | Tyrone | 2010 | | | 1079 | | Sitharama Iyengar | | S. | | 1998 | |
| 1040 | N. Srivastava | | Ashok | 2010 | | | 1080 | | Kim | | Kane | | 1998 | |
| 1041 | Zhang | | Hong-Jiang | 2010 | | | 1081 | | W. Wah | | Benjamin | | 1998 | |
| 1042 | Govindaraju | | Venu | 2010 | | | 1082 | | Luqi | |  | | 1997 | |
| 1043 | Santos | | Eunice | 2010 | | | 1083 | | J.P. Tsai | | Jeffrey | | 1997 | |
| 1044 | Ferrari | | Elena | 2009 | | | 1084 | | Thuraisingham | | Bhavani | | 1997 | |
| 1045 | S. Bay | | John | 2009 | | | 1085 | | Aggarwal | | J.K. | | 1996 | |
| 1046 | K. Das | | Sajal | 2009 | | | 1086 | | Ranjan Das | | Sunil | | 1996 | |
| 1047 | W. Finin | | Timothy | 2009 | | | 1087 | | Patterson | | David | | 1995 | |
| 1048 | Frieder | | Ophir | 2008 | | | 1088 | | Feng | | Tse-yun | | 1991 | |
| 1049 | Chellapa | | Rama | 2008 | | | 1089 | | R. Boggs | | David | | 1988 | |
| 1050 | S. Trivedi | | Kishor | 2008 | | | 1090 | | M. Metcalfe | | Robert | | 1987 | |
| 1051 | Tamassia | | Roberto | 2006 | | | 1091 | | Iwasaki | | S. | | 1987 | |
| 1052 | T. Goodrich | | Michael | 2006 | | | 1092 | | A. Avizienis | | Algirdas | | 1985 | |
| 1053 | Shekhar | | Shashi | 2006 | | | 1093 | | J. McCluskey | | Edward | | 1985 | |
| 1054 | Chen | | Hsinchun | 2006 | | |  | |  | |  | |  | |
| 1055 | E. Cooke | | Daniel | 2005 | | |  | |  | |  | |  | |
| 1056 | A. Forsyth | | David | 2005 | | |  | |  | |  | |  | |
| 1057 | Mukherjee | | Amar | 2005 | | |  | |  | |  | |  | |
| 1058 | Kumar | | Vipin | 2005 | | |  | |  | |  | |  | |
|  |  | |  |  | | |  | |  | |  | |  | |

| **SOFTWARE SYSTEM AWARD** | | | | | | | |
| --- | --- | --- | --- | --- | --- | --- | --- |
| **Sr. #** | **First name** | **Last Name** | **year** | **Sr. #** | **First name** | **Last Name** | **year** |
| 1094 | Greg | Adams | 2011 | 1133 | Lee R | Gordon | 2010 |
| 1095 | Vikram | Adve | 2012 | 1134 | James | Gosling | 2002 |
| 1096 | Marc | Andreessen | 1995 | 1135 | Goetz | Graefe | 2008 |
| 1097 | Bruno | Barras | 2013 | 1136 | Jim | Gray | 1988 |
| 1098 | Brian | Behlendorf | 1999 | 1137 | Kevin | Haaland | 2011 |
| 1099 | Peter | Bergstrom | 2010 | 1138 | David | Harel | 2007 |
| 1100 | Tim | Berners-Lee | 1995 | 1139 | Rob | Hartill | 1999 |
| 1101 | Yves | Bertot | 2013 | 1140 | Gerald | Held | 1988 |
| 1102 | Eric | Bina | 1995 | 1141 | Hugo | Herbelin | 2013 |
| 1103 | Raghuram | Bindignavle | 2004 | 1142 | Jonathan L | Herlocker | 2010 |
| 1104 | Andrew | Birrell | 1994 | 1143 | Michael L | Heytens | 2008 |
| 1105 | Daniel | Bobrow | 1992 | 1144 | Gerard J. | Holzmann | 2001 |
| 1106 | Robert S | Boyer | 2005 | 1145 | Hui-I | Hsiao | 2008 |
| 1107 | Daniel S | Bricklin | 1985 | 1146 | Gerard P | Huet | 2013 |
| 1108 | Douglas K | Brotz | 1989 | 1147 | Neophytos | Iacovou | 2010 |
| 1109 | Edouard | Bugnion | 2009 | 1148 | Daniel H.H. | Ingalls | 1987 |
| 1110 | Richard R | Burton | 1992 | 1149 | Julian | Jones | 2011 |
| 1111 | Robert | Cailliau | 1995 | 1150 | Robert E | Kahn | 1991 |
| 1112 | Pierre | Castéran | 2013 | 1151 | Ronald M. | Kaplan | 1992 |
| 1113 | Vinton | Cerf | 1991 | 1152 | Matt | Kaufmann | 2005 |
| 1114 | Donald | Chamberlin | 1988 | 1153 | Alan | Kay | 1987 |
| 1115 | John M. | Chambers | 1998 | 1154 | Donald E | Knuth | 1986 |
| 1116 | Evan | Cheng | 2012 | 1155 | Joseph A | Konstan | 2010 |
| 1117 | Thierry | Coquand | 2013 | 1156 | Murali | Krishna | 2008 |
| 1118 | L Peter | Deutsch | 1992 | 1157 | Hagi | Lachover | 2007 |
| 1119 | Scott | Devine | 2009 | 1158 | Shyong K. | Lam | 2010 |
| 1120 | David | DeWitt | 2008 | 1159 | Simon | Lam | 2004 |
| 1121 | John | Duimovich | 2011 | 1160 | Butler W | Lampson | 1984 |
| 1122 | Douglas | Engelbart | 1990 | 1161 | Chris | Lattner | 2012 |
| 1123 | William K. | English | 1990 | 1162 | Raymond A | Lorie | 1988 |
| 1124 | Stuart | Feldman | 2003 | 1163 | David | Maltz | 2010 |
| 1125 | Roy T. | Fielding | 1999 | 1164 | Larry M | Masinter | 1992 |
| 1126 | Jean-Christophe | Filliâtre | 2013 | 1165 | Sean | McNee | 2010 |
| 1127 | Robert | Frankston | 1985 | 1166 | Bertrand | Meyer | 2006 |
| 1128 | Erich | Gamma | 2011 | 1167 | Bradley N | Miller | 2010 |
| 1129 | Robert | Gerber | 2008 | 1168 | J Strother | Moore | 2005 |
| 1130 | Charles M | Geschke | 1989 | 1169 | Philippe | Mulet | 2011 |
| 1131 | Shahram | Ghandeharizadeh | 2008 | 1170 | Chetan | Murthy | 2013 |
| 1132 | Adele | Goldberg | 1987 | 1171 | Amnon | Naamad | 2007 |
|  |  |  |  |  |  |  |  |
| **Sr. #** | **First name** | **Last Name** | **year** |  |  |  |  |
| 1172 | Jeffrey F | Naughton | 2008 |  |  |  |  |
| 1173 | Bruce | Nelson | 1994 |  |  |  |  |
| 1174 | Steve | Northover | 2011 |  |  |  |  |
| 1175 | John K | Ousterhout | 1997 |  |  |  |  |
| 1176 | Christine | Paulin-Mohring | 2013 |  |  |  |  |
| 1177 | William H. | Paxton | 1989 |  |  |  |  |
| 1178 | Amir | Pnueli | 2007 |  |  |  |  |
| 1179 | Michal | Politi | 2007 |  |  |  |  |
| 1180 | Gianfranco | Putzolu | 1988 |  |  |  |  |
| 1181 | Paul J | Resnick | 2010 |  |  |  |  |
| 1182 | John T | Riedl | 2010 |  |  |  |  |
| 1183 | Dennis M. | Ritchie | 1983 |  |  |  |  |
| 1184 | David | Robinson | 1999 |  |  |  |  |
| 1185 | Mendel | Rosenblum | 2009 |  |  |  |  |
| 1186 | Jeff | Rulifson | 1990 |  |  |  |  |
| 1187 | Donovan A | Schneider | 2008 |  |  |  |  |
| 1188 | Patricia | Selinger | 1988 |  |  |  |  |
| 1189 | Anoop | Sharma | 2008 |  |  |  |  |
| 1190 | Rivi | Sherman | 2007 |  |  |  |  |
| 1191 | Cliff | Skolnick | 1999 |  |  |  |  |
| 1192 | Michael | Stonebraker | 1988 |  |  |  |  |
| 1193 | Shaowen | Su | 2004 |  |  |  |  |
| 1194 | Mitesh | Suchak | 2010 |  |  |  |  |
| 1195 | Jeremy | Sugerman | 2009 |  |  |  |  |
| 1196 | Ivan | Sutherland | 1993 |  |  |  |  |
| 1197 | Edward A. | Taft | 1989 |  |  |  |  |
| 1198 | Robert W | Taylor | 1984 |  |  |  |  |
| 1199 | Warren | Teitelman | 1992 |  |  |  |  |
| 1200 | Randy | Terbush | 1999 |  |  |  |  |
| 1201 | Charles P | Thacker | 1984 |  |  |  |  |
| 1202 | Robert S. | Thau | 1999 |  |  |  |  |
| 1203 | Kenneth Lane | Thompson | 1983 |  |  |  |  |
| 1204 | Dave | Thomson | 2011 |  |  |  |  |
| 1205 | Irv | Traiger | 1988 |  |  |  |  |
| 1206 | Mark | Trakhtenbrot | 2007 |  |  |  |  |
| 1207 | Aron | Trauring | 2007 |  |  |  |  |
| 1208 | Ellen | Wang | 2009 |  |  |  |  |
| 1209 | John | Warnock | 1989 |  |  |  |  |
| 1210 | John | Weigand | 2011 |  |  |  |  |
| 1211 | Andrew | Wilson | 1999 |  |  |  |  |
| 1212 | Eugene | Wong | 1988 |  |  |  |  |
| 1213 | Thomas Y. C. | Woo | 2004 |  |  |  |  |

| **Gordon Bell Prize** | | | | | | | | | | | | | | | | | | |
| --- | --- | --- | --- | --- | --- | --- | --- | --- | --- | --- | --- | --- | --- | --- | --- | --- | --- | --- |
| **Sr. #** | **First name** | | **Last Name** | | **year** | **Sr. #** | | **First name** | | | **Last Name** | | | | | **year** | | |
| 1214 | Nikolaus | | Adams | | 2013 | 1255 | | George | | | Karniadakis | | | | | 2011 | | |
| 1215 | Costas | | Bekas | | 2013 | 1256 | | Gerhard | | | Klimeck | | | | | 2011 | | |
| 1216 | Adam | | Bertsch | | 2013 | 1257 | | Kalyan | | | Kumaran | | | | | 2011 | | |
| 1217 | Alessandro | | Curioni | | 2013 | 1258 | | Mathieu | | | Luisier | | | | | 2011 | | |
| 1218 | Scott | | Futral | | 2013 | 1259 | | Satoshi | | | Matsuoka | | | | | 2011 | | |
| 1219 | Panagiotis | | Hadjidoukas | | 2013 | 1260 | | Simone | | | Melchionna | | | | | 2011 | | |
| 1220 | Babak | | Hejazialhosseini | | 2013 | 1261 | | Vitali A | | | Morozov | | | | | 2011 | | |
| 1221 | Petros | | Koumoutsakos | | 2013 | 1262 | | Michael | | | Papka | | | | | 2011 | | |
| 1222 | Diego | | Rossinelli | | 2013 | 1263 | | Suro | | | Succi | | | | | 2011 | | |
| 1223 | Steffen | | Schmidt | | 2013 | 1264 | | George | | | Biros | | | | | 2010 | | |
| 1224 | Tomoaki | | Ishiyama | | 2012 | 1265 | | Aparna | | | Chandramowlishwaran | | | | | 2010 | | |
| 1225 | Keigo | | Nitadori | | 2012 | 1266 | | Ilya | | | Lashuk | | | | | 2010 | | |
| 1226 | Junichiro | | Makino | | 2012 | 1267 | | Dhairya | | | Malhotra | | | | | 2010 | | |
| 1227 | Taisuke | | Boku | | 2011 | 1268 | | Logan | | | Moon | | | | | 2010 | | |
| 1228 | Yukihiro | | Hasegawa | | 2011 | 1269 | | Abtin | | | Rahimian | | | | | 2010 | | |
| 1229 | Junichi | | Iwata | | 2011 | 1270 | | Rahul | | | Sampath | | | | | 2010 | | |
| 1230 | Motoyoshi | | Kurokawa | | 2011 | 1271 | | Aashay | | | Shringarpure | | | | | 2010 | | |
| 1231 | Kazuo | | Minami | | 2011 | 1272 | | Shravan | | | Veerapaneni | | | | | 2010 | | |
| 1232 | Atsushi | | Oshiyama | | 2011 | 1273 | | Jeffrey S | | | Vetter | | | | | 2010 | | |
| 1233 | Fumiyoshi | | Shoji | | 2011 | 1274 | | Richard W. | | | Vuduc | | | | | 2010 | | |
| 1234 | Daisuke | | Takahashi | | 2011 | 1275 | | Denis | | | Zorin | | | | | 2010 | | |
| 1235 | Miwako | | Tsuji | | 2011 | 1276 | | Adolfo G. | | | Eguiluz | | | | | 2010 | | |
| 1236 | Atsuya | | Uno | | 2011 | 1277 | | Anton | | | Kozhevnikov | | | | | 2010 | | |
| 1237 | Mitsuo | | Yokokawa | | 2011 | 1278 | | Thomas | | | Schulthess | | | | | 2010 | | |
| 1238 | Takayuki | | Aoki | | 2011 | 1279 | | Tsuyoshi | | | Hamada | | | | | 2010 | | |
| 1239 | Toshio | | Endo | | 2011 | 1280 | | Keigo | | | Nitadori | | | | | 2010 | | |
| 1240 | Naoya | | Maruyama | | 2011 | 1281 | | Tetsu | | | Narumi | | | | | 2009 | | |
| 1241 | Satoshi | | Matsuoka | | 2011 | 1282 | | Keigo | | | Nitadori | | | | | 2009 | | |
| 1242 | Akira | | Nukada | | 2011 | 1283 | | Kiyoshi | | | Oguri | | | | | 2009 | | |
| 1243 | Takashi | | Shimokawabe | | 2011 | 1284 | | Makoto | | | Taiji | | | | | 2009 | | |
| 1244 | Tomohiro | | Takaki | | 2011 | 1285 | | Kenji | | | Yasuoka | | | | | 2009 | | |
| 1245 | Akinori | | Yamanaka | | 2011 | 1286 | | Rio | | | Yokota | | | | | 2009 | | |
| 1246 | Massimo | | Bernaschi | | 2011 | 1287 | | Rajapopal | | | Ananthanarayanan | | | | | 2009 | | |
| 1247 | Mauro | | Bisson | | 2011 | 1288 | | Joseph A. | | | Bank | | | | | 2009 | | |
| 1248 | Timothy B. | | Boykin | | 2011 | 1289 | | Brannon | | | Batson | | | | | 2009 | | |
| 1249 | Toshio | | Endo | | 2011 | 1290 | | Kevin J. | | | Bowers | | | | | 2009 | | |
| 1250 | Massimiliano | | Fatica | | 2011 | 1291 | | Edmond | | | Chow | | | | | 2009 | | |
| 1251 | Dimitry | | Fedosov | | 2011 | 1292 | | Martin M. | | | Deneroff | | | | | 2009 | | |
| 1252 | Wolfgang | | Fichtner | | 2011 | 1293 | | Ron | | | Dror | | | | | 2009 | | |
| 1253 | Leopold | | Grinberg | | 2011 | 1294 | | Michael P. | | | Eastwood | | | | | 2009 | | |
| 1254 | Joseph A | | Insley | | 2011 | 1295 | | Steven K. | | | Esser | | | | | 2009 | | |
| **Sr. #** | **First name** | | **Last Name** | | **year** | **Sr. #** | | **First name** | | | **Last Name** | | | | | **year** | | |
| 1296 | J.P. | | Grossman | | 2009 | 1339 | | John | | | Gunnels | | | | | 2007 | | |
| 1297 | Doug John | | Ierardi | | 2009 | 1340 | | David F | | | Richards | | | | | 2007 | | |
| 1298 | John L. | | Klepeis | | 2009 | 1341 | | Robert E | | | Rudd | | | | | 2007 | | |
| 1299 | Jeffrey | | Kuskin | | 2009 | 1342 | | Frederick H | | | Streitz | | | | | 2007 | | |
| 1300 | Richard | | Larson | | 2009 | 1343 | | Vernon | | | Austel | | | | | 2006 | | |
| 1301 | Kresten | | Lindorff-Larsen | | 2009 | 1344 | | Bronis R. | | | De Supinski | | | | | 2006 | | |
| 1302 | Kenneth M. | | Mackenzie | | 2009 | 1345 | | Erik W. | | | Draeger | | | | | 2006 | | |
| 1303 | Paul | | Maragakis | | 2009 | 1346 | | Franz | | | Franchetti | | | | | 2006 | | |
| 1304 | Dharmendra | | Modha | | 2009 | 1347 | | John A. | | | Gunnels | | | | | 2006 | | |
| 1305 | Mark | | Moraes | | 2009 | 1348 | | Francois | | | Gygi | | | | | 2006 | | |
| 1306 | Stefano | | Piana | | 2009 | 1349 | | Stefan | | | Kral | | | | | 2006 | | |
| 1307 | John | | Salmon | | 2009 | 1350 | | Juergen | | | Lorenz | | | | | 2006 | | |
| 1308 | Yibing | | Shan | | 2009 | 1351 | | Martin | | | Schulz | | | | | 2006 | | |
| 1309 | David | | Shaw | | 2009 | 1352 | | James C. | | | Sexton | | | | | 2006 | | |
| 1310 | Horst D | | Simon | | 2009 | 1353 | | Christoph W | | | Ueberhuber | | | | | 2006 | | |
| 1311 | Brian | | Towles | | 2009 | 1354 | | Gyan | | | Bhanot | | | | | 2006 | | |
| 1312 | Cliff | | Young | | 2009 | 1355 | | Matt | | | Blumrich | | | | | 2006 | | |
| 1313 | Gregory | | Brown | | 2009 | 1356 | | Dong | | | Chen | | | | | 2006 | | |
| 1314 | Markus | | Eisenbach | | 2009 | 1357 | | Alan | | | Gara | | | | | 2006 | | |
| 1315 | Jeffrey M. | | Larkin | | 2009 | 1358 | | Mark | | | Giampapa | | | | | 2006 | | |
| 1316 | Donald M. | | Nicholson | | 2009 | 1359 | | Philip | | | Heidelberger | | | | | 2006 | | |
| 1317 | Thomas | | Schulthess | | 2009 | 1360 | | Valentina | | | Salapura | | | | | 2006 | | |
| 1318 | Chenggang | | Zhou | | 2009 | 1361 | | James C. | | | Sexton | | | | | 2006 | | |
| 1319 | Gonzalo | | Alvarez | | 2008 | 1362 | | Ron | | | Soltz | | | | | 2006 | | |
| 1320 | Eduardo Francisco | | D'Azevedo | | 2008 | 1363 | | Pavlos | | | Vranas | | | | | 2006 | | |
| 1321 | Markus | | Eisenbach | | 2008 | 1364 | | Noriyuki | | | Fatatsugi | | | | | 2006 | | |
| 1322 | Paul | | Kent | | 2008 | 1365 | | Shigenori | | | Fujikawa | | | | | 2006 | | |
| 1323 | Jeffrey M. | | Larkin | | 2008 | 1366 | | Ryutaro | | | Himeno | | | | | 2006 | | |
| 1324 | John M. | | Levesque | | 2008 | 1367 | | Mitsuru | | | Ikei | | | | | 2006 | | |
| 1325 | Thomas A. | | Maier | | 2008 | 1368 | | Takahiro | | | Koishi | | | | | 2006 | | |
| 1326 | Don E. | | Maxwell | | 2008 | 1369 | | Tetsu | | | Narumi | | | | | 2006 | | |
| 1327 | Jeremy | | Meredith | | 2008 | 1370 | | Yousuke | | | Ohno | | | | | 2006 | | |
| 1328 | Thomas | | Schulthess | | 2008 | 1371 | | Noriaki | | | Okimoto | | | | | 2006 | | |
| 1329 | Michael S. | | Summers | | 2008 | 1372 | | Atsushi | | | Suenaga | | | | | 2006 | | |
| 1330 | David H | | Bailey | | 2008 | 1373 | | Makoto | | | Taiji | | | | | 2006 | | |
| 1331 | Byounghak | | Lee | | 2008 | 1374 | | Ryoko | | | Yanai | | | | | 2006 | | |
| 1332 | Juan | | Meza | | 2008 |  | |  | | |  | | | | |  | | |
| 1333 | Hongzhang | | Shan | | 2008 |  | |  | | |  | | | | |  | | |
| 1334 | Erich | | Strohmaier | | 2008 |  | |  | | |  | | | | |  | | |
| 1335 | Lin-Wang | | Wang | | 2008 |  | |  | | |  | | | | |  | | |
| 1336 | Zhengji | | Zhao | | 2008 |  | |  | | |  | | | | |  | | |
| 1337 | Kyle J | | Caspersen | | 2007 |  | |  | | |  | | | | |  | | |
| 1338 | James N | | Glosli | | 2007 |  | |  | | |  | | | | |  | | |
| **COMPUTER PIONEER AWARD** | | | | | | | | | | | | | | | | |  |  |
| **Sr. #** | | **First name** | | **Last Name** | **year** | | **Sr. #** | | **First name** | | | **Last Name** | | **year** | | |  |  |
| 1375 | | Torvalds | | Linus | 2014 | | 1415 | | | C. Moisil | | | Grigore | | 1996 | | |  |
| 1376 | | B. Furber | | Stephen | 2013 | | 1416 | | | Plander | | | Ivan | | 1996 | | |  |
| 1377 | | Feigenbaum | | Edward | 2013 | | 1417 | | | Reitsakas | | | Arnols | | 1996 | | |  |
| 1378 | | Moler | | Cleve | 2012 | | 1418 | | | Svoboda | | | Antonin | | 1996 | | |  |
| 1379 | | Kuck | | David | 2011 | | 1419 | | | Estrin | | | Gerald | | 1995 | | |  |
| 1380 | | Sammet | | Jean | 2009 | | 1420 | | | Evans | | | David | | 1995 | | |  |
| 1381 | | Conway | | Lynn | 2009 | | 1421 | | | Lampson | | | Butler | | 1995 | | |  |
| 1382 | | Jean Jennings Bartik | | Betty | 2008 | | 1422 | | | Minsky | | | Marvin | | 1995 | | |  |
| 1383 | | J. McCluskey | | Edward | 2008 | | 1423 | | | Olsen | | | Kenneth | | 1995 | | |  |
| 1384 | | A. Petri | | Carl | 2008 | | 1424 | | | A. Blaauw | | | Gerrit | | 1994 | | |  |
| 1385 | | Hosaka | | Mamoru | 2006 | | 1425 | | | D. Mills | | | Harlan | | 1994 | | |  |
| 1386 | | M. Spielberg | | Arnold | 2006 | | 1426 | | | M. Ritchie | | | Dennis | | 1994 | | |  |
| 1387 | | (Fran) E. Allen | | Frances | 2004 | | 1427 | | | L. Thompson | | | Ken | | 1994 | | |  |
| 1388 | | Richards | | Martin | 2003 | | 1428 | | | Bloch | | | Erich | | 1993 | | |  |
| 1389 | | Brinch Hansen | | Per | 2002 | | 1429 | | | S. Kilby | | | Jack | | 1993 | | |  |
| 1390 | | W. Bemer | | Robert | 2002 | | 1430 | | | H. Ware | | | Willis | | 1993 | | |  |
| 1391 | | L. Schatz | | Vernon | 2001 | | 1431 | | | W. Dunwell | | | Stephen | | 1992 | | |  |
| 1392 | | H. Bridge | | William | 2001 | | 1432 | | | C. Engelbart | | | Douglas | | 1992 | | |  |
| 1393 | | W. Lawson | | Harold | 2000 | | 1433 | | | O. Evans | | | Bob | | 1991 | | |  |
| 1394 | | Stolyarov | | Gennady | 2000 | | 1434 | | | W. Floyd | | | Robert | | 1991 | | |  |
| 1395 | | Lopato | | Georgiy | 2000 | | 1435 | | | E. Kurtz | | | Thomas | | 1991 | | |  |
| 1396 | | Freeman | | Herbert | 1999 | | 1436 | | | Buchholz | | | Werner | | 1990 | | |  |
| 1397 | | John (Jack) Good | | Irving | 1998 | | 1437 | | | Hoare | | | C.A.R. | | 1990 | | |  |
| 1398 | | (Barney) Oldfield | | Homer | 1997 | | 1438 | | | Cocke | | | John | | 1989 | | |  |
| 1399 | | Elizabeth (Betty) Snyder-Holberton | | Frances | 1997 | | 1439 | | | A. Weidenhammer | | | James | | 1989 | | |  |
| 1400 | | Angelov | | Angel | 1996 | | 1440 | | | L. Palmer | | | Ralph | | 1989 | | |  |
| 1401 | | F. Clippinger | | Richard | 1996 | | 1441 | | | S. Rees | | | Mina | | 1989 | | |  |
| 1402 | | Frank Codd | | Edgar | 1996 | | 1442 | | | C. Yovits | | | Marshall | | 1989 | | |  |
| 1403 | | Fristacky | | Norbert | 1996 | | 1443 | | | Joachim Weyl | | | F. | | 1989 | | |  |
| 1404 | | M. Glushkov | | Victor | 1996 | | 1444 | | | D. Goldstein | | | Gordon | | 1989 | | |  |
| 1405 | | Gruska | | Jozef | 1996 | | 1445 | | | L. Bauer | | | Freidrich | | 1988 | | |  |
| 1406 | | Horejs | | Jiri | 1996 | | 1446 | | | E. Hoff, Jr. | | | Marcian | | 1988 | | |  |
| 1407 | | Georgiev Iliev | | Lubomir | 1996 | | 1447 | | | R. Everett | | | Robert | | 1987 | | |  |
| 1408 | | E. Kahn | | Robert | 1996 | | 1448 | | | B. Johnson | | | Reynold | | 1987 | | |  |
| 1409 | | Kalmar | | Laszlo | 1996 | | 1449 | | | L. Samuel | | | Arthur | | 1987 | | |  |
| 1410 | | Kilinski | | Antoni | 1996 | | 1450 | | | E. Wirth | | | Nicklaus | | 1987 | | |  |
| 1411 | | Kozma | | Laszlo | 1996 | | 1451 | | | C. Hurd | | | Cuthbert | | 1986 | | |  |
| 1412 | | A. Lebedev | | Sergey | 1996 | | 1452 | | | Naur | | | Peter | | 1986 | | |  |
| 1413 | | A. Lyapunov | | Alexey | 1996 | | 1453 | | | H. Pomerene | | | James | | 1986 | | |  |
| 1414 | | W. Marczynski | | Romuald | 1996 | | 1454 | | | van Wijngaarden | | | Adriann | | 1986 | | |  |
| **Sr. #** | | **First name** | | **Last Name** | **year** | |  | | |  | | |  | |  | | |  |
| 1455 | | G. Kemeny | | John | 1985 | |  | | |  | | |  | |  | | |  |
| 1456 | | McCarthy | | John | 1985 | |  | | |  | | |  | |  | | |  |
| 1457 | | Perlis | | Alan | 1985 | |  | | |  | | |  | |  | | |  |
| 1458 | | Sutherland | | Ivan | 1985 | |  | | |  | | |  | |  | | |  |
| 1459 | | J. Wheeler | | David | 1985 | |  | | |  | | |  | |  | | |  |
| 1460 | | Zemanek | | Heinz | 1985 | |  | | |  | | |  | |  | | |  |
| 1461 | | Vincent Atanasoff | | John | 1984 | |  | | |  | | |  | |  | | |  |
| 1462 | | A. Haddad | | Jerrier | 1984 | |  | | |  | | |  | |  | | |  |
| 1463 | | C. Metropolis | | Nicholas | 1984 | |  | | |  | | |  | |  | | |  |
| 1464 | | Rochester | | Nathaniel | 1984 | |  | | |  | | |  | |  | | |  |
| 1465 | | L. van der Poel | | Willem | 1984 | |  | | |  | | |  | |  | | |  |
| 1466 | | D. Huskey | | Harry | 1982 | |  | | |  | | |  | |  | | |  |
| 1467 | | Burks | | Arthur | 1982 | |  | | |  | | |  | |  | | |  |
| 1468 | | Chuan Chu | | Jeffrey | 1981 | |  | | |  | | |  | |  | | |  |

| **IEEE JONH VON NEUMANN AWARD** | | | | | | | |
| --- | --- | --- | --- | --- | --- | --- | --- |
| **Sr. #** | **First name** | **Last Name** | **year** | **Sr. #** | **First name** | **Last Name** | **year** |
| 1469 | A. Gosling | James | 2015 | 1492 | E. Knuth | Donald | 1995 |
| 1470 | Moler | Cleve | 2014 | 1493 | Cocke | John | 1994 |
| 1471 | Dennis | Jack | 2013 | 1494 | P. Brooks | Frederick | 1993 |
| 1472 | J. McCluskey | Edward | 2012 | 1495 | Gordon Bell | C. | 1992 |
| 1473 | A. R. Hoare | C. | 2011 |  |  |  |  |
| 1474 | Ullman | Jeffrey | 2010 |  |  |  |  |
| 1475 | Hopcroft | John | 2010 |  |  |  |  |
| 1476 | L. Graham | Susan | 2009 |  |  |  |  |
| 1477 | Lamport | Leslie | 2008 |  |  |  |  |
| 1478 | P. Thacker | Charles | 2007 |  |  |  |  |
| 1479 | Catmull | Edwin | 2006 |  |  |  |  |
| 1480 | Stonebraker | Michael | 2005 |  |  |  |  |
| 1481 | H. Liskov | Barbara | 2004 |  |  |  |  |
| 1482 | V. Aho | Alfred | 2003 |  |  |  |  |
| 1483 | Nygaard | Kristen | 2002 |  |  |  |  |
| 1484 | Dahl | Ole-Johan | 2002 |  |  |  |  |
| 1485 | W. Lampson | Butler | 2001 |  |  |  |  |
| 1486 | A. Patterson | David | 2000 |  |  |  |  |
| 1487 | L. Hennessy | John | 2000 |  |  |  |  |
| 1488 | C. Engelbart | Douglas | 1999 |  |  |  |  |
| 1489 | Edward Sutherland | Ivan | 1998 |  |  |  |  |
| 1490 | V. Wilkes | Maurice | 1997 |  |  |  |  |
| 1491 | A. Mead | Carver | 1996 |  |  |  |  |

| **INFOSYS FOUNDATION AWARD** | | | | | Programming Languages Achievement Award | | | |
| --- | --- | --- | --- | --- | --- | --- | --- | --- |
| **Sr. #** | **First name** | **Last Name** | | **year** | **Sr. #** | **First name** | **Last Name** | **year** |
| 1496 | Sanjeev | Arora | | 2011 | 1520 | D. Jones | Neil | 2014 |
| 1497 | David M. | Blei | | 2013 | 1521 | Cousot | Radhia | 2013 |
| 1498 | Eric A. | Brewer | | 2009 | 1522 | Cousot | Patrick | 2013 |
| 1499 | Jeffrey A | Dean | | 2012 | 1523 | Felleisen | Matthias | 2012 |
| 1500 | Sanjay | Ghemawat | | 2012 | 1524 | Hoare | Tony | 2011 |
| 1501 | Frans | Kaashoek | | 2010 | 1525 | Plotkin | Gordon | 2010 |
| 1502 | Jon | Kleinberg | | 2008 | 1526 | Burstall | Rod | 2009 |
| 1503 | Daphne | Koller | | 2007 | 1527 | Liskov | Barbara | 2008 |
| **SEYMOUR CRAY AWARD** | | | | | 1528 | Wirth | Niklaus | 2007 |
| **Sr. #** | **First name** | | **Last Name** | **year** | 1529 | Cytron | Ron | 2006 |
| 1504 | Bell | | Gordon | 2014 | 1530 | Ferrante | Jeanne | 2006 |
| 1505 | Snir | | Marc | 2013 | 1531 | Helm | Richard | 2005 |
| 1506 | M. Kogge | | Peter | 2012 | 1532 | Gamma | Erich | 2005 |
| 1507 | L. Seitz | | Charles | 2011 | 1533 | Backus | John | 2004 |
| 1508 | Gara | | Alan | 2010 | 1534 | C. Reynolds | John | 2003 |
| 1509 | Miura | | Kenichi | 2009 | 1535 | McCarthy | John | 2002 |
| 1510 | Wallach | | Steve | 2008 | 1536 | Milner | Robin | 2001 |
| 1511 | E. Batcher | | Kenneth | 2007 | 1537 | Graham | Susan | 2000 |
| 1512 | Watanabe | | Tadashi | 2006 | 1538 | Kennedy | Ken | 1999 |
| 1513 | L. Scott | | Steven | 2005 | 1539 | Allen | Fran | 1998 |
| 1514 | J. Dally | | William | 2004 | 1540 | Steele | Guy | 1997 |
| 1515 | J. Smith | | Burton | 2003 |  |  |  |  |
| 1516 | M. Denneau | | Monty | 2002 |  |  |  |  |
| 1517 | L. Hennessy | | John | 2001 |  |  |  |  |
| 1518 | J. Culler | | Glen | 2000 |  |  |  |  |
| 1519 | Cocke | | John | 1999 |  |  |  |  |

| **DISTINGUISHED SERVICE AWARD** | | | | | | | |
| --- | --- | --- | --- | --- | --- | --- | --- |
| **Sr. #** | **First name** | **Last Name** | **year** | **Sr. #** | **First name** | **Last Name** | **year** |
| 1541 | Gerhard T | Goos | 2013 | 1550 | Susan L | Graham | 2006 |
| 1542 | Juris | Hartmanis | 2013 | 1551 | Mary Jane | Irwin | 2005 |
| 1543 | Jan | van Leeuwen | 2013 | 1552 | Ed | Coffman | 2004 |
| 1544 | Mateo | Valero | 2012 | 1553 | Ruzena R | Bajcsy | 2003 |
| 1545 | William A | Wulf | 2011 | 1554 | Raymond | Miller | 2002 |
| 1546 | Reinhard | Wilhelm | 2010 | 1555 | Won | Kim | 2001 |
| 1547 | Edward | Lazowska | 2009 | 1556 | Peter | Wegner | 2000 |
| 1548 | Telle | Whitney | 2008 | 1557 | Anita | Borg | 1999 |
| 1549 | David | Patterson | 2007 | 1558 | David H | Brandin | 1997 |
| **Sr. #** | **First name** | **Last Name** | **year** | **Sr. #** | **First name** | **Last Name** | **year** |
| 1559 | Hal | Berghel | 1996 | 1572 | Anthony | Ralston | 1982 |
| 1560 | Doris | Lidtke | 1995 | 1573 | Aaron | Finerman | 1981 |
| 1561 | J.A.N. | Lee | 1993 | 1574 | Bernard | Galler | 1980 |
| 1562 | Joyce Currie | Little | 1992 | 1575 | Carl | Hammer | 1979 |
| 1563 | Gerald L | Engel | 1991 | 1576 | Eric A. | Weiss | 1978 |
| 1564 | Walter | Carlson | 1990 | 1577 | Thomas B | Steel | 1977 |
| 1565 | Peter J | Denning | 1989 | 1578 | Richard G. | Canning | 1976 |
| 1566 | Charles L | Bradshaw | 1988 | 1579 | John W. | Carr III | 1975 |
| 1567 | Frederick | Brooks | 1987 | 1580 | Saul | Gorn | 1974 |
| 1568 | Clair | Maple | 1986 | 1581 | William F. | Atchison | 1973 |
| 1569 | Jean E | Sammet | 1985 | 1582 | George E. | Forsythe | 1972 |
| 1570 | Saul | Rosen | 1984 | 1583 | J. Don | Madden | 1971 |
| 1571 | Grace Murray | Hopper | 1983 | 1584 | Franz L | Alt | 1970 |

| **DOCTORAL DISSERTATION AWARD** | | | | | | | |
| --- | --- | --- | --- | --- | --- | --- | --- |
| **Sr. #** | **First name** | **Last Name** | **year** | **Sr. #** | **First name** | **Last Name** | **year** |
| 1585 | Grey | Ballard | 2013 | 1607 | Aseem | Agarwala | 2006 |
| 1586 | Sanjam | Garg | 2013 | 1608 | Yi-Ren | Ng | 2006 |
| 1587 | Shayan Oveis | Gharan | 2013 | 1609 | Olivier | Dousse | 2005 |
| 1588 | Shyamnath | Gollakota | 2012 | 1610 | Ben | Liblit | 2005 |
| 1589 | Peter | Hawkins | 2012 | 1611 | Boaz | Barak | 2004 |
| 1590 | Gregory | Valiant | 2012 | 1612 | Ramesh | Johari | 2004 |
| 1591 | Seth | Cooper | 2011 | 1613 | Emmett | Witchel | 2004 |
| 1592 | Aleksander | Madry | 2011 | 1614 | AnHai | Doan | 2003 |
| 1593 | David | Steurer | 2011 | 1615 | Dina | Katabi | 2003 |
| 1594 | Bryan | Parno | 2010 | 1616 | Subhash | Khot | 2003 |
| 1595 | Benjamin | Snyder | 2010 | 1617 | Venkatesan | Guruswami | 2002 |
| 1596 | Craig | Gentry | 2009 | 1618 | Robert C. | Miller | 2002 |
| 1597 | Haryadi S | Gunawi | 2009 | 1619 | Tim | Roughgarden | 2002 |
| 1598 | Andre | Platzer | 2009 | 1620 | Robert | O'Callahan | 2001 |
| 1599 | Keith Noah | Snavely | 2009 | 1621 | Ion | Stoica | 2001 |
| 1600 | Constantinos | Daskalakis | 2008 | 1622 | David | Wagner | 2001 |
| 1601 | Derek | Hoiem | 2008 | 1623 | William | Chan | 2000 |
| 1602 | Sachin | Katti | 2008 | 1624 | Michael D. | Ernst | 2000 |
| 1603 | Benny | Applebaum | 2007 | 1625 | Salil P | Vadhan | 2000 |
| 1604 | Vincent | Conitzer | 2007 | 1626 | Dieter | van Melkebeek | 1999 |
| 1605 | Yan | Liu | 2007 | 1627 | Hari | Balakrishnan | 1998 |
| 1606 | Sergey | Yekhanin | 2007 | 1628 | Steven R. | McCanne | 1997 |
| **Sr. #** | **First name** | **Last Name** | **year** | **Sr. #** | **First name** | **Last Name** | **year** |
| 1629 | Xiaoyuan | Tu | 1996 | 1653 | Marc H. | Brown | 1987 |
| 1630 | Carl | Waldspurger | 1996 | 1654 | John | Canny | 1987 |
| 1631 | Sanjeev | Arora | 1995 | 1655 | Leslie | Greengard | 1987 |
| 1632 | Daniel A | Spielman | 1995 | 1656 | Carl | Ebeling | 1986 |
| 1633 | David | Karger | 1994 | 1657 | Ketan D. | Mulmuley | 1986 |
| 1634 | T.V. | Raman | 1994 | 1658 | Johan | Torkel Hastad | 1986 |
| 1635 | James J. | Kistler | 1993 | 1659 | David M | Ungar | 1986 |
| 1636 | Pandu | Nayak | 1993 | 1660 | Ben-Zion | Chor | 1985 |
| 1637 | Madhu | Sudan | 1993 | 1661 | John R. | Ellis | 1985 |
| 1638 | Kenneth | McMillan | 1992 | 1662 | William Daniel | Hillis | 1985 |
| 1639 | Mendel | Rosenblum | 1992 | 1663 | Carl E. | Bach | 1984 |
| 1640 | Asit | Dan | 1991 | 1664 | Henry | Baird | 1984 |
| 1641 | Garth A | Gibson | 1991 | 1665 | Manolis G.H. | Katevenis | 1984 |
| 1642 | Carsten | Lund | 1991 | 1666 | James | Korein | 1984 |
| 1643 | Robert | Schapire | 1991 | 1667 | Ellen | Hildreth | 1983 |
| 1644 | Hector | Geffner | 1990 | 1668 | Steven | Johnson | 1983 |
| 1645 | David | Heckerman | 1990 | 1669 | Thomas | Reps | 1983 |
| 1646 | Noam | Nissan | 1990 | 1670 | Charles E | Leiserson | 1982 |
| 1647 | Michael J. | Kearns | 1989 | 1671 | Douglas | Cook | 1980 |
| 1648 | Joe | Killian | 1989 | 1672 | Ruth E. | Davis | 1980 |
| 1649 | Vijay | Saraswat | 1989 | 1673 | Lawrence Edwin | Larson | 1980 |
| 1650 | Anne | Condon | 1988 | 1674 | Jacob | Slonim | 1980 |
| 1651 | David | Dill | 1988 | 1675 | Roderic G. | Cattell | 1978 |
| 1652 | Mauricio | Karchmer | 1988 | 1676 | Joseph | Urban | 1978 |

| IEEE CS ECKERT-MAUCHLY AWARD | | | | | | | |
| --- | --- | --- | --- | --- | --- | --- | --- |
| **Sr. #** | **First name** | **Last Name** | **year** | **Sr. #** | **First name** | **Last Name** | **year** |
| 1677 | Trevor | Mudge | 2014 | 1684 | Mateo | Valero | 2007 |
| 1678 | James | Goodman | 2013 | 1685 | James H | Pomerene | 2006 |
| 1679 | Algirdas | Avizienis | 2012 | 1686 | Robert P. | Colwell | 2005 |
| 1680 | Gurindar S | Sohi | 2011 | 1687 | Frederick | Brooks | 2004 |
| 1681 | William J | Dally | 2010 | 1688 | Joseph A. (Josh) | Fisher | 2003 |
| 1682 | Joel | Emer | 2009 | 1689 | Bantwal R | Rau | 2002 |
| 1683 | David | Patterson | 2008 | 1690 | John L | Hennessy | 2001 |
| **Sr. #** | **First name** | **Last Name** | **year** | **Sr. #** | **First name** | **Last Name** | **year** |
| 1689 | Bantwal R | Rau | 2002 | 1701 | Kenneth E. | Batcher | 1990 |
| 1690 | John L | Hennessy | 2001 | 1702 | Seymour | Cray | 1989 |
| 1691 | Edward | Davidson | 2000 | 1703 | Daniel | Siewiorek | 1988 |
| 1692 | James E. | Smith | 1999 | 1704 | Gene M. | Amdahl | 1987 |
| 1693 | T. | Watanabe | 1998 | 1705 | Harvey G | Cragon | 1986 |
| 1694 | Robert | Tomasulo | 1997 | 1706 | John | Cocke | 1985 |
| 1695 | Yale | Patt | 1996 | 1707 | Jack | Dennis | 1984 |
| 1696 | John | Crawford | 1995 | 1708 | Tom | Kilburn | 1983 |
| 1697 | James E. | Thornton | 1994 | 1709 | C Gordon | Bell | 1982 |
| 1698 | David J | Kuck | 1993 | 1710 | Wesley A. | Clark | 1981 |
| 1699 | Michael J | Flynn | 1992 | 1711 | Maurice V. | Wilkes | 1980 |
| 1700 | Burton J | Smith | 1991 | 1712 | Robert S. | Barton | 1979 |

| Grace Murray Hopper Award | | | | | | | |
| --- | --- | --- | --- | --- | --- | --- | --- |
| **Sr. #** | **First name** | **Last Name** | **year** | **Sr. #** | **First name** | **Last Name** | **year** |
| 1713 | Pedro F | Felzenszwalb | 2013 | 1733 | William Daniel | Hillis | 1989 |
| 1714 | Martin | Casado | 2012 | 1734 | Guy L | Steele | 1988 |
| 1715 | Dina | Katabi | 2012 | 1735 | John K | Ousterhout | 1987 |
| 1716 | Luis | Von Ahn | 2011 | 1736 | William N. | Joy | 1986 |
| 1717 | Craig | Gentry | 2010 | 1737 | Cordell | Green | 1985 |
| 1718 | Tim | Roughgarden | 2009 | 1738 | Daniel H.H. | Ingalls | 1984 |
| 1719 | Dawson | Engler | 2008 | 1739 | Brian K. | Reid | 1982 |
| 1720 | Vern | Paxson | 2007 | 1740 | Daniel S | Bricklin | 1981 |
| 1721 | Daniel | Klein | 2006 | 1741 | Robert M. | Metcalfe | 1980 |
| 1722 | Omer | Reingold | 2005 | 1742 | Stephen | Wozniak | 1979 |
| 1723 | Jennifer | Rexford | 2004 | 1743 | Ray | Kurzweil | 1978 |
| 1724 | Stephen | Keckler | 2003 | 1744 | Edward H | Shortliffe | 1976 |
| 1725 | Ramakrishnan | Srikant | 2002 | 1745 | Allan L. | Scherr | 1975 |
| 1726 | George | Necula | 2001 | 1746 | George N. | Baird | 1974 |
| 1727 | Lydia | Kavraki | 2000 | 1747 | Lawrence | Breed | 1973 |
| 1728 | Wen-Mei | Hwu | 1999 | 1748 | Richard | Lathwell | 1973 |
| 1729 | Shafi | Goldwasser | 1996 | 1749 | Roger | Moore | 1973 |
| 1730 | Bjarne | Stroustrup | 1993 | 1750 | Paul H. | Cress | 1972 |
| 1731 | Feng-hsiung | Hsu | 1991 | 1751 | Paul E. | Dirksen | 1972 |
| 1732 | Richard | Stallman | 1990 | 1752 | Donald E | Knuth | 1971 |

| Paris Kanellakis Theory and Practice award | | | | | | | |
| --- | --- | --- | --- | --- | --- | --- | --- |
| **Sr. #** | **First name** | **Last Name** | **year** | **Sr. #** | **First name** | **Last Name** | **year** |
| 1753 | Robert | Blumofe | 2013 | 1774 | Robert | Solovay | 2003 |
| 1754 | Charles E | Leiserson | 2013 | 1775 | Volker | Strassen | 2003 |
| 1755 | Andrei | Broder | 2012 | 1776 | Peter A. | Franaszek | 2002 |
| 1756 | Moses S | Charikar | 2012 | 1777 | Eugene | Myers | 2001 |
| 1757 | Piotr | Indyk | 2012 | 1778 | Narendra | Karmarkar | 2000 |
| 1758 | Hanan | Samet | 2011 | 1779 | Daniel | Sleator | 1999 |
| 1759 | Kurt | Mehlhorn | 2010 | 1780 | Robert E | Tarjan | 1999 |
| 1760 | Mihir | Bellare | 2009 | 1781 | Randal E | Bryant | 1998 |
| 1761 | Phillip | Rogaway | 2009 | 1782 | Edmund | Clarke | 1998 |
| 1762 | Corinna | Cortes | 2008 | 1783 | E. Allen | Emerson | 1998 |
| 1763 | Vladimir | Vapnik | 2008 | 1784 | Kenneth L. | McMillan | 1998 |
| 1764 | Bruno | Buchberger | 2007 | 1785 | Abraham | Lempel | 1997 |
| 1765 | Robert | Brayton | 2006 | 1786 | Jacob | Ziv | 1997 |
| 1766 | Gerard J. | Holzmann | 2005 | 1787 | Leonard M. | Adleman | 1996 |
| 1767 | Robert P. | Kurshan | 2005 | 1788 | Whitfield | Diffie | 1996 |
| 1768 | Moshe Y | Vardi | 2005 | 1789 | Martin | Hellman | 1996 |
| 1769 | Pierre | Wolper | 2005 | 1790 | Ralph | Merkle | 1996 |
| 1770 | Yoav | Freund | 2004 | 1791 | Ronald L | Rivest | 1996 |
| 1771 | Robert | Schapire | 2004 | 1792 | Adi | Shamir | 1996 |
| 1772 | Gary L | Miller | 2003 |  |  |  |  |
| 1773 | Michael O. | Rabin | 2003 |  |  |  |  |

| Karl V. Karlstrom Outstanding educator Award | | | | | | | | |
| --- | --- | --- | --- | --- | --- | --- | --- | --- |
| **Sr. #** | | **First name** | **Last Name** | **year** | **Sr. #** | **First name** | **Last Name** | **year** |
| 1793 | Susan | | Rodger | 2013 | 1799 | John E | Hopcroft | 2008 |
| 1794 | Eric S | | Roberts | 2012 | 1800 | Randy | Pausch | 2007 |
| 1795 | Hal | | Abelson | 2011 | 1801 | Stuart J | Russell | 2005 |
| 1796 | Barbara | | Ericson | 2010 | 1802 | Sartaj K | Sahni | 2003 |
| 1797 | Mark | | Guzdial | 2010 | 1803 | John | Gorgone | 2002 |
| 1798 | Matthias | | Felleisen | 2009 |  |  |  |  |
|  |  | |  |  |  |  |  |  |
| **Sr. #** | **First name** | | **Last Name** | **year** | **Sr. #** | **First name** | **Last Name** | **year** |
| 1804 | Nell B. | | Dale | 2001 | 1811 | Andrew S. | Tanenbaum | 1994 |
| 1805 | Yale | | Patt | 2000 | 1812 | Andries | van Dam | 1993 |
| 1806 | Randy H. | | Katz | 1999 | 1813 | David | Harel | 1992 |
| 1807 | Abraham | | Silberschatz | 1998 | 1814 | David | Patterson | 1991 |
| 1808 | Jeffrey D | | Ullman | 1997 | 1815 | Gerald | Sussman | 1990 |
| 1809 | Peter J | | Denning | 1996 | 1816 | C.L. | Liu | 1989 |
| 1810 | David | | Gries | 1995 |  |  |  |  |

| Eugene L. Lawler Award | | | | | | | | |
| --- | --- | --- | --- | --- | --- | --- | --- | --- |
| **Sr. #** | **First name** | **Last Name** | **year** |  |  |  |  |  |
| 1817 | Thomas | Bartoschek | 2012 |  |  |  |  |  |
| 1818 | Johannes | Schoening | 2012 |  |  |  |  |  |
| 1819 | Gregory | Abowd | 2009 |  |  |  |  |  |
| 1820 | Randy | Wang | 2007 |  |  |  |  |  |
| 1821 | Albrecht | Ehrensperger | 2005 |  |  |  |  |  |
| 1822 | Solomon | Mbuguah | 2005 |  |  |  |  |  |
| 1823 | Ernest | Siva | 2005 |  |  |  |  |  |
| 1824 | Patrick | Ball | 2003 |  |  |  |  |  |
| 1825 | John | Blitch | 2001 |  |  |  |  |  |
| 1826 | Antonia | Stone | 1999 |  |  |  |  |  |
| IEEE CS George Michael HPC Fellowships | | | | | | | | |
| 1827 | Alexander | Breuer | 2014 | 1843 | Abhinav | Bhatele | 2009 |  |
| 1828 | Harshitha | Menon | 2014 | 1844 | Amanda | Randles | 2009 |  |
| 1829 | Jonathan | Lifflander | 2013 | 1845 | Mark | Silberstein | 2009 |  |
| 1830 | Edgar | Solomonik | 2013 | 1846 | Nathan | Tallent | 2009 |  |
| 1831 | Ryan | Gabrys | 2012 | 1847 | Yong | Chen | 2008 |  |
| 1832 | Gagan | Gupta | 2012 | 1848 | Sean M. | Couch | 2008 |  |
| 1833 | Amanda | Randles | 2012 | 1849 | Yaniv | Erlich | 2008 |  |
| 1834 | Yanhua | Sun | 2012 | 1850 | Samer Al | Kiswany | 2008 |  |
| 1835 | Leonardo Arturo | Bautista Gomez | 2011 | 1851 | Douglas | Mason | 2008 |  |
| 1836 | Michael J. | Duchene | 2011 | 1852 | Daniel J | Quest | 2008 |  |
| 1837 | Ignacio | Laguna | 2011 | 1853 | Yong | Chen | 2007 |  |
| 1838 | Xinyu | Que | 2011 | 1854 | Mark | Hoemmen | 2007 |  |
| 1839 | Sara | Baghsorkhi | 2010 | 1855 | Arpith | Jacob | 2007 |  |
| 1840 | Aparna | Chandramowlishwaran | 2010 | 1856 | Kamesh | Madduri | 2007 |  |
| 1841 | Matthew R. | Norman | 2010 | 1857 | Chao | Wang | 2007 |  |
| 1842 | Amanda | Randles | 2010 |  |  |  |  |  |

| Outstanding Countribution to ACM Award | | | | | | | |
| --- | --- | --- | --- | --- | --- | --- | --- |
| **Sr. #** | **First name** | **Last Name** | **year** | **Sr. #** | **First name** | **Last Name** | **year** |
| 1858 | Donna | Cappo | 2013 | 1881 | John H. (Jack) | Esbin | 1993 |
| 1859 | Russell | Harris | 2013 | 1882 | Frank L | Friedman | 1993 |
| 1860 | Zvi | Kedem | 2012 | 1883 | James M. | Adams | 1992 |
| 1861 | Calvin C. | Gotlieb | 2011 | 1884 | Lorraine | Borman | 1992 |
| 1862 | Joseph S | DeBlasi | 2010 | 1885 | Peter G | Neumann | 1992 |
| 1863 | Moshe Y | Vardi | 2009 | 1886 | Allen | Tucker | 1991 |
| 1864 | Wayne | Graves | 2008 | 1887 | William B | Poucher | 1990 |
| 1865 | Bernard | Rous | 2008 | 1888 | Monroe | Newborn | 1989 |
| 1866 | Robert A. | Walker | 2007 | 1889 | Thomas | DeFanti | 1988 |
| 1867 | David S | Wise | 2006 | 1890 | Ed | Coffman | 1987 |
| 1868 | Don | Gotterbarn | 2005 | 1891 | Herbert | Maisel | 1986 |
| 1869 | Richard T | Snodgrass | 2004 | 1892 | Thomas A. | D'Auria | 1985 |
| 1870 | Mark Scott | Johnson | 2003 | 1893 | Jack | Minker | 1985 |
| 1871 | Pat | Ryan | 2002 | 1894 | Orrin E. | Taulbee | 1984 |
| 1872 | Barbara B | Simons | 2001 | 1895 | Richard H | Austing | 1983 |
| 1873 | Hal | Berghel | 2000 | 1896 | Seymour J | Wolfson | 1983 |
| 1874 | Ronald | Boisvert | 1999 | 1897 | Fred H | Harris | 1982 |
| 1875 | R L | Ashenhurst | 1998 | 1898 | J.A.N. | Lee | 1981 |
| 1876 | Peter J | Denning | 1998 | 1899 | M Stuart | Lynn | 1979 |
| 1877 | Stuart | Zweben | 1997 | 1900 | Kathleen A. | Wagner | 1978 |
| 1878 | Robert M | Aiken | 1996 | 1901 | W. Smith | Dorsey | 1976 |
| 1879 | A. Joe | Turner | 1995 | 1902 | Bruce W. | Van Atta | 1976 |
| 1880 | John R. | White | 1994 |  |  |  |  |

| AAAI Allen Newell Award | | | | | | | |
| --- | --- | --- | --- | --- | --- | --- | --- |
| **Sr. #** | **First name** | **Last Name** | **year** | **Sr. #** | **First name** | **Last Name** | **year** |
| 1903 | Yoav | Shoham | 2012 | 1913 | Richard | Gabriel | 2004 |
| 1904 | Moshe | Tennenholtz | 2012 | 1914 | David | Haussler | 2003 |
| 1905 | Stephanie | Forrest | 2011 | 1915 | Judea | Pearl | 2003 |
| 1906 | Takeo | Kanade | 2010 | 1916 | Peter | Chen | 2002 |
| 1907 | Michael I. | Jordan | 2009 | 1917 | Ruzena R | Bajcsy | 2001 |
| 1908 | Barbara J | Grosz | 2008 | 1918 | Lotfi | Zadeh | 2000 |
| 1909 | Joseph | Halpern | 2008 | 1919 | Nancy | Leveson | 1999 |
| 1910 | Leonidas J | Guibas | 2007 | 1920 | Saul | Amarel | 1998 |
| 1911 | Karen | Sparck-Jones | 2006 | 1921 | Carver | Mead | 1997 |
| 1912 | Jack | Minker | 2005 | 1922 | Joshua | Lederberg | 1995 |
|  |  |  |  | 1923 | Frederick | Brooks | 1994 |

| **ACM Presedential Award** | | | | | | | |
| --- | --- | --- | --- | --- | --- | --- | --- |
| **Sr. #** | **First name** | **Last Name** | **year** | **Sr. #** | **First name** | **Last Name** | **year** |
| 1924 | Mehran | Sahami | 2014 | 1933 | Moshe Y | Vardi | 2008 |
| 1925 | Fabrizio | Gagliardi | 2013 | 1934 | Eugene H | Spafford | 2006 |
| 1926 | Yunhao | Liu | 2013 | 1935 | Andreas | Bechtolsheim | 2005 |
| 1927 | P J | Narayanan | 2013 | 1936 | Edward | Lazowska | 2005 |
| 1928 | Mathai | Joseph | 2010 | 1937 | Calvin C. | Gotlieb | 2002 |
| 1929 | Elaine J | Weyuker | 2010 | 1938 | John | Gage | 1998 |
| 1930 | Stephen | Bourne | 2008 | 1939 | Kent K. | Curtis | 1987 |
| 1931 | Pat | Ryan | 2008 | 1940 | Robert E | Kahn | 1985 |
| 1932 | Barbara G | Ryder | 2008 |  |  |  |  |

| ACM prize in comp. science and Engg. | | | | | | | |
| --- | --- | --- | --- | --- | --- | --- | --- |
| **Sr. #** | **First name** | **Last Name** | **year** | **Sr. #** | **First name** | **Last Name** | **year** |
| 1941 | Satish | Balay | 2014 | 1948 | Linda | Petzold | 2013 |
| 1942 | Jed | Brown | 2014 | 1949 | J. Tinsley | Oden | 2011 |
| 1943 | William D | Gropp | 2014 | 1950 | Cleve | Moler | 2009 |
| 1944 | Matthew | Knepley | 2014 | 1951 | Chi-Wang | Shu | 2007 |
| 1945 | Lois C | McInnes | 2014 | 1952 | Achi | Brandt | 2005 |
| 1946 | Barry | Smith | 2014 | 1953 | John B. | Bell | 2002 |
| 1947 | Hong | Zhang | 2014 | 1954 | Phillip | Colella | 2002 |
| Programming system and language award | | | | | | | |
| **Sr. #** | **First name** | **Last Name** | **year** | **Sr. #** | **First name** | **Last Name** | **year** |
| 1955 | Michael W. | Blasgen | 1982 | 1970 | Kenneth Lane | Thompson | 1975 |
| 1956 | Jim | Gray | 1982 | 1971 | Daniel | Bobrow | 1974 |
| 1957 | Bruce | Lindsay | 1982 | 1972 | Zohar | Manna | 1974 |
| 1958 | Raymond A | Lorie | 1982 | 1973 | Stephen | Ness | 1974 |
| 1959 | Paul R | McJones | 1982 | 1974 | Jean | Vuillemin | 1974 |
| 1960 | Thomas G | Price | 1982 | 1975 | Ben | Wegbreit | 1974 |
| 1961 | Gianfranco | Putzolu | 1982 | 1976 | C. Antony R. | Hoare | 1973 |
| 1962 | Irv | Traiger | 1982 | 1977 | Peter J | Denning | 1971 |
| 1963 | David Lorge | Parnas | 1979 | 1978 | Edsger W | Dijkstra | 1971 |
| 1964 | Niklaus E | Wirth | 1978 | 1979 | Jan | Gecsei | 1971 |
| 1965 | David | Gries | 1977 | 1980 | Peter | Lucas | 1971 |
| 1966 | Susan S | Owicki | 1977 | 1981 | Richard L | Mattson | 1971 |
| 1967 | Frances | Allen | 1976 | 1982 | John C | Reynolds | 1971 |
| 1968 | John | Cocke | 1976 | 1983 | Donald R | Slutz | 1971 |
| 1969 | Dennis M. | Ritchie | 1975 | 1984 | Irv | Traiger | 1971 |
|  |  |  |  | 1985 | Kurt | Walk | 1971 |

| **ACM-W Athena Lecture Award** | | | |
| --- | --- | --- | --- |
| **Sr. #** | **First name** | **Last Name** | **year** |
| 1986 | Susan T | Dumais | 2014 |
| 1987 | Kathy | Yelick | 2013 |
| 1988 | Nancy A | Lynch | 2012 |
| 1989 | Judith S | Olson | 2011 |
| 1990 | Mary Jane | Irwin | 2010 |
| 1991 | Susan | Eggers | 2009 |
| 1992 | Shafi | Goldwasser | 2008 |
| 1993 | Karen | Sparck-Jones | 2007 |
| 1994 | Deborah | Estrin | 2006 |
